# Direct Assembly of Metal‐Phenolic Network Nanoparticles for Biomedical Applications

**DOI:** 10.1002/anie.202312925

**Published:** 2023-10-06

**Authors:** Wanjun Xu, Zhixing Lin, Shuaijun Pan, Jingqu Chen, Tianzheng Wang, Christina Cortez‐Jugo, Frank Caruso

**Affiliations:** ^1^ Department of Chemical Engineering The University of Melbourne Parkville Victoria 3010 Australia; ^2^ State Key Laboratory of Chemo/Biosensing and Chemometrics and College of Chemistry and Chemical Engineering Hunan University Changsha 410082 China

**Keywords:** Biomedicine, Coordination Assembly, Metal-Organic Networks, Nanoparticles, Polyphenols

## Abstract

Coordination assembly offers a versatile means to developing advanced materials for various applications. However, current strategies for assembling metal‐organic networks into nanoparticles (NPs) often face challenges such as the use of toxic organic solvents, cytotoxicity because of synthetic organic ligands, and complex synthesis procedures. Herein, we directly assemble metal‐organic networks into NPs using metal ions and polyphenols (i.e., metal‐phenolic networks (MPNs)) in aqueous solutions without templating or seeding agents. We demonstrate the role of buffers (e.g., phosphate buffer) in governing NP formation and the engineering of the NP physicochemical properties (e.g., tunable sizes from 50 to 270 nm) by altering the assembly conditions. A library of MPN NPs is prepared using natural polyphenols and various metal ions. Diverse functional cargos, including anticancer drugs and proteins with different molecular weights and isoelectric points, are readily loaded within the NPs for various applications (e.g., biocatalysis, therapeutic delivery) by direct mixing, without surface modification, owing to the strong affinity of polyphenols to various guest molecules. This study provides insights into the assembly mechanism of metal‐organic complexes into NPs and offers a simple strategy to engineer nanosized materials with desired properties for diverse biotechnological applications.

## Graphical abstract

Metal‐phenolic network nanoparticles (MPN NPs) with different compositions are directly assembled, without templates or seeding agents, by controlling the coordination kinetics of metal‐phenolic complexes. The strong binding affinity of polyphenols to various guest molecules enables the incorporation of functional cargos (e.g., proteins and drugs) to form cargo‐loaded MPN NPs for potential biomedical applications.

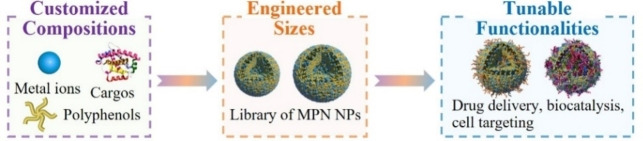

## Introduction

Exploiting coordination‐based assembly for the rational design of advanced materials is of fundamental interest in diverse fields.^[1]^ In particular, the assembly of metal‐organic networks, formed through the coordination of metal ions and organic ligands, into nanoparticles (NPs) has attracted widespread interest owing to their potential for environmental, agricultural, and biomedical applications.^[2]^ Different strategies, such as rapid nucleation (e.g., “initiation‐solvent” approach), confined space (e.g., nanoemulsions), and coordination modulation (e.g., stabilizing agents), have been developed to fabricate metal‐organic network NPs.^[3]^ However, these approaches often use nonbiocompatible synthetic ligands and/or require harsh synthesis conditions (e.g., organic solvents or high temperature),^[4a]^ thereby in some cases limiting their practical applications and posing potential environmental and health risks.^[4b]^ Moreover, the adoption of case‐by‐case synthesis protocols, which is required because of different coordination states, and the limited availability of synthetic ligands further restrict the development of metal‐organic network NPs.^[5]^ Therefore, there remains a need for a simple and broadly applicable methodology that can produce NPs from a wide range of available ligands and metal ions for various applications.

Polyphenols are abundant in nature with over 6000 known organic species that can coordinate with metal ions to form metal‐phenolic network (MPN) coatings on various substrates (e.g., organic, inorganic, and biological entities).^[6]^ The rapid chelation between phenolic ligands and metal ions enables the formation of MPN coatings within minutes in aqueous conditions;^[7]^ however, the rapid coordination kinetics also impedes their further formation into well‐defined NPs. Accordingly, seeding agents (e.g., synthetic polymers) and templates have been used to regulate metal‐phenolic complexes and facilitate NP formation.^[8]^ However, the addition of seeding agents can alter the NP properties (e.g., pH responsiveness) and the use of templates requires additional steps. Alternatively, the direct synthesis of MPN NPs without seeding agents or templates is desirable though challenging.[^3a^, ^4b^] Moreover, the (direct) assembly of such MPN NPs could inspire the rational design of other metal‐organic NP systems.

**Figure 1:**
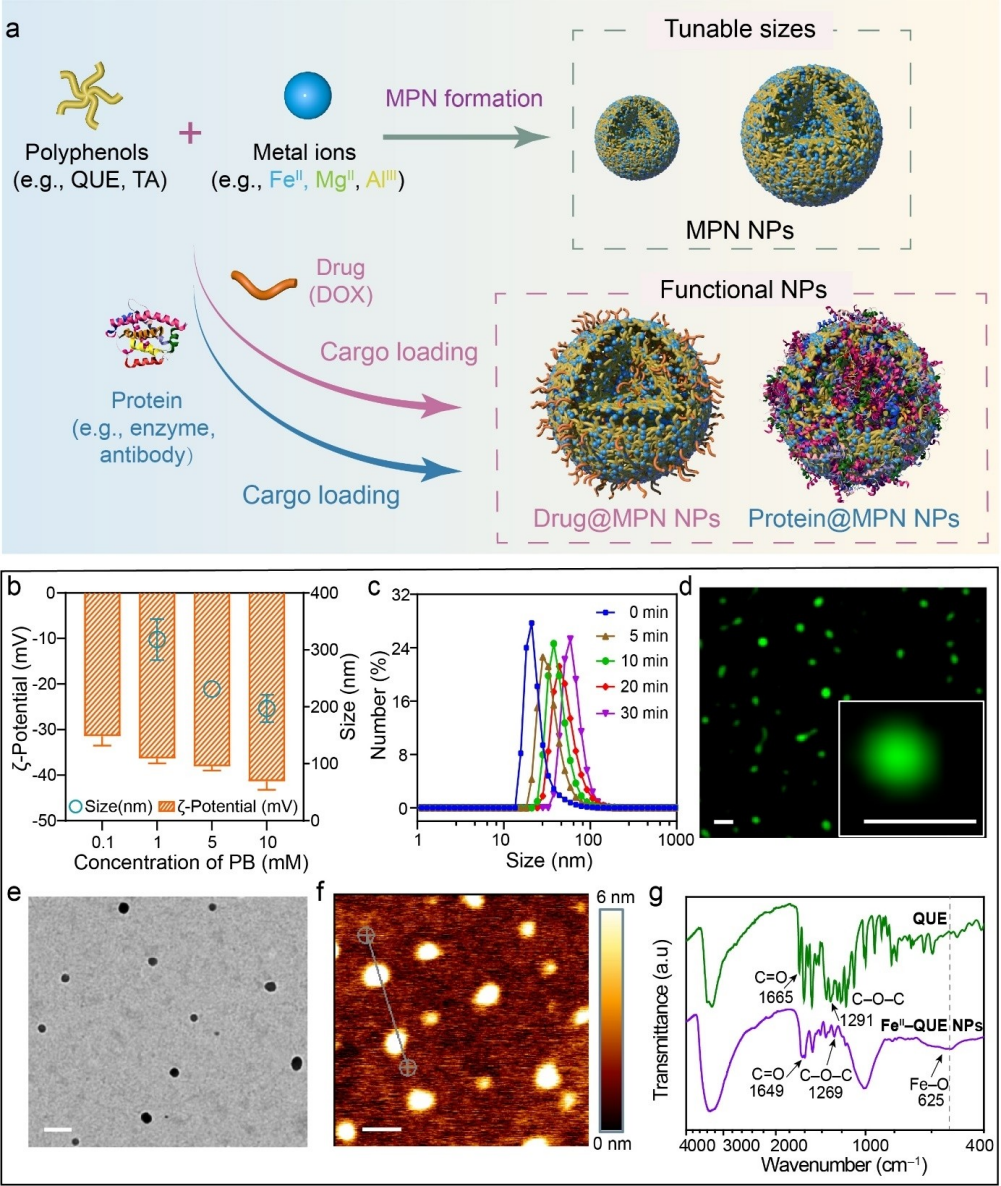
a) Schematic of the direct assembly of MPN NPs and cargo‐loaded MPN NPs. b) Size, as determined by DLS, and ζ‐potential values of MPN NPs assembled using different concentrations of PB. Note that 0 and 0.1 mM PB did not result in MPN NP formation. Data are shown as the mean ± standard deviation (SD) (*n*=3). c) Time‐dependent DLS data, showing the growth of MPN NPs. d) Super‐resolution lattice SIM images of FITC‐labeled MPN NPs. Scale bars are 200 nm. e) TEM image of MPN NPs. Scale bar is 200 nm. f) AFM image of MPN NPs. Cross section given in Figure S8. Scale bar is 200 nm. g) FTIR spectra of QUE and MPN (Fe^II^‐QUE) NPs.

Herein, we report the direct assembly of a library of MPN NPs composed only of phenolic ligands (with single or multimodal coordination sites) and metal ions with different valences (Figure 1a). The formation mechanism and kinetics of MPN NPs were examined, and the coordination modes of the MPN NPs were engineered by altering the assembly conditions (e.g., assembly pH, reaction time, precursor concentration), leading to NPs with tunable physicochemical properties (e.g., size, shape, and surface chemistry). The MPN NPs were stable under simulated physiological environments and exhibited high biocompatibility. In addition, the strong binding affinity of polyphenols to various guest molecules enabled the incorporation of functional cargos, including small molecule drugs and proteins with different molecular weights and isoelectric points (pIs), into the MPN NPs, which have potential applications in cell targeting, biocatalysis, and therapeutic delivery. This strategy expands the realm of MPN materials by creating a NP library for diverse applications and is expected to underpin the rational assembly of other metal‐organic NP systems.

## Results and Discussion

The formation mechanism of MPN NPs (without templates or seeding agents) was first investigated by mixing metal ions and polyphenols in various buffer solutions with the same pH at ambient temperature (i.e., 22 °C). Fe^II^ and quercetin (QUE), a natural polyphenol found in many plants and foods, were selected as the model metal ion and ligand, respectively, for the fabrication of MPN NPs.^[9]^ MPN NP formation was observed in phosphate buffer (PB) and citrate buffer but not in non‐ or weakly coordinating buffers (e.g., 3‐(N‐morpholino)propanesulfonic acid and 2‐(N‐morpholino)ethanesulfonic acid (MES)), which are commonly used in MPN studies (Figures S1 and S2).^[10]^ This suggests that interactions between metal ions and buffers plays an essential role in mediating the coordination kinetics and modes of metal‐phenolic complexes, thus influencing NP formation (Figure S3).^[11]^ For example, in PB, the Fe^II^‐QUE MPNs that could form NPs featured a higher proportion of Fe‐maltol and tris‐state Fe‐catechol coordination and a lower proportion of bis‐state and mono‐state Fe‐catechol coordination when compared with the degree of the coordination modes observed in MES buffer, which did not result in NP formation (Figure S3d). The relatively stronger interaction of the metal ions with coordinating buffers (e.g., PB) results in a higher extent of cross‐linked and more stable coordination networks, which may facilitate nucleation and thus NP formation.^[12]^ Moreover, increased concentrations of PB (from 1 to 10 mM) led to the formation of smaller NPs (from ≈320 to ≈175 nm), likely due to a higher number of NPs formed as a result of a larger number of nuclei formed at a given precursor concentration (Figure 1b, Table S1). Furthermore, the size of the MPN NPs was controlled by adjusting the reaction time, precursor concentration, and metal‐to‐ligand ratio. Time‐dependent dynamic light scattering (DLS) results showed that the size of MPN NPs gradually increased from 20 to 60 nm as the reaction time increased from 0 to 30 min and then plateaued, likely due to depletion of the precursors (Figure 1c, Figure S4). When the concentrations of the precursors were simultaneously increased ([Fe^II^] from 1 to 10 mg mL^−1^ and [QUE] from 0.5 to 5 mg mL^−1^) while keeping a constant Fe^II^‐to‐QUE ratio of 8 : 1, the size of the MPN NPs increased from ≈50 to ≈160 nm (Figure S5). Small‐angle X‐ray scattering was used to elucidate the mechanism underlying the concentration‐dependent NP size changes. As observed from Figure S6, a higher building block concentration resulted in a higher scattering intensity at a small scattering vector *q*, corresponding to the formation of larger NPs. The size of the NPs could also be increased (from 50 to 150 nm) by increasing the Fe^II^‐to‐QUE molar ratio from 0.5 to 16 (Figure S7).

Optical microscopy and electron microscopy were used to characterize the structure and coordination states of the MPN NPs, which were assembled from 10 mg mL^−1^ Fe^II^ and 5 mg mL^−1^ QUE. Super‐resolution lattice structured illumination microscopy (SIM) showed the fluorescein isothiocyanate (FITC)‐labeled MPN NPs are essentially spherical and have a diameter of 150±18 nm in aqueous solution (Figure 1d). Transmission electron microscopy (TEM) showed spherical NPs (in an air‐dried state), while atomic force microscopy (AFM) images displayed collapsed NPs, with a height of ≈5 nm, following air drying (Figure 1e, f and Figure S8). Fourier transform infrared (FTIR) spectroscopy showed the presence of an Fe−O stretching vibration band at 625 cm^−1^, indicating the occurrence of metal coordination in the MPN NPs. Additionally, the formation of metal coordination resulted in hypochromic shifts of the aryl ketonic stretching band (C=O; from 1665 to 1649 cm^−1^) and the aromatic ester stretching band (C−O−C; from 1291 to 1269 cm^−1^) (Figure 1g).^[13]^

**Figure 2:**
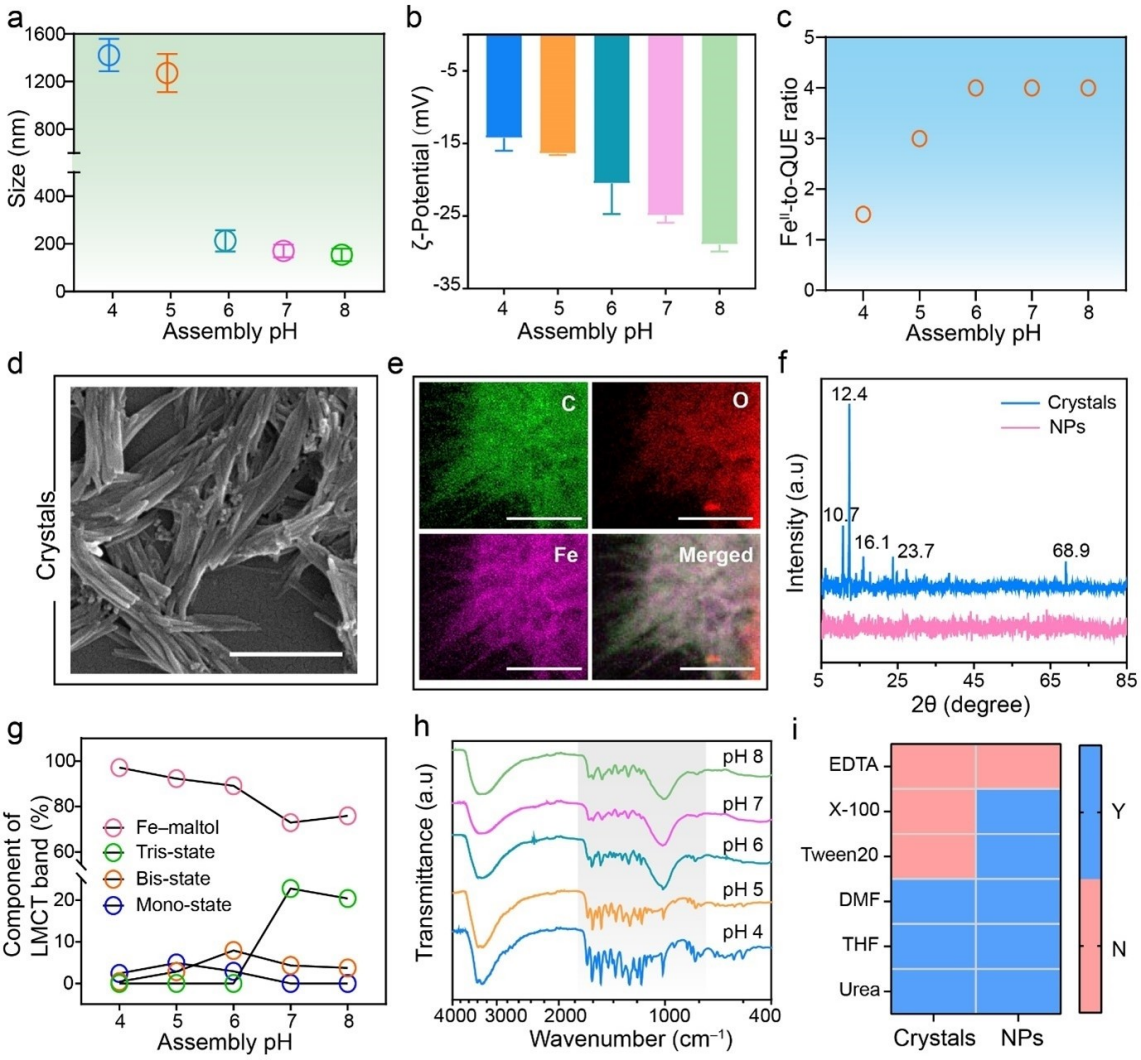
a–c) Size, as determined by DLS (a), ζ‐potential values (b), and Fe^II^‐to‐QUE ratio (c) of the MPN particles assembled at various pH values. Data are shown as the mean±SD (*n*=3). d) SEM image and e) EDX elemental mapping of MPN crystals obtained at pH 4. Scale bars are 1 μm. f) XRD patterns of MPN crystals and NPs. g) Percentages of Fe‐maltol, and tri‐state, bis‐state, and mono‐state Fe‐catechol coordination of the MPN particles as a function of assembly pH. h) FTIR spectra of MPN particles assembled at different pH values. i) Heatmap illustrating the stability of MPN crystals and NPs upon incubating in different media. EDTA, ethylenediaminetetraacetic acid; X‐100, Triton X‐100; DMF, dimethylformamide; THF, tetrahydrofuran. Y means stable and N means disassembled.

We next investigated the effect of assembly pH on the formation and physiochemical properties of the MPN NPs, as coordination assemblies are often dynamic and influenced by pH.^[14]^ As observed from Figure 2a, as the assembly pH increased from 4 to 8, a transition in the formation of larger particles (≈1400 nm) to NPs (≈150 nm) was obtained. Given that QUE has an acid dissociation constant (p*K*_a_) of 6.4,^[15]^ the larger particles observed at the lower pH values (e.g., pH 4 and 5) can be attributed to the aggregation of QUE molecules due to the likely increased π–π and hydrophobic interactions of protonated QUE. The deprotonation/protonation of QUE also resulted in a transition in ζ‐potential from −15 to −30 mV within the pH range of 4 to 8 (Figure 2b). Additionally, the assembly pH influenced the Fe^II^‐to‐QUE ratio in the formed MPN particles (Figure 2c). Scanning electron microscopy (SEM) and AFM images showed that the MPN particles prepared at low pH (e.g., pH 4) exhibited a needle‐like (acicular) shape with a thickness of approximately 40 nm (Figure 2d, Figure S9). Energy‐dispersive X‐ray spectroscopy (EDX) elemental mapping revealed a uniform distribution of elements C, O, and Fe within the needle‐like shape, confirming the presence of Fe^II^‐QUE networks (Figure 2e). The sharp X‐ray diffraction (XRD) patterns demonstrated the high degree of crystallinity of the needle‐like MPN particles (crystals) assembled at pH 4, whereas no obvious peaks (i.e., amorphous) were observed in the MPN NPs assembled at pH 7 (Figure 2f). In addition, Fourier transforms of the X‐ray absorption spectroscopy data of the MPN crystals and NPs showed similar dominant peaks of Fe−O coordination bond within the 1–2 Å region, whereas the 1s→3d transition featuring at 7112 eV for the crystals was 1.25‐fold more intense than that of the NPs (Figure S10), suggesting distinct coordination modes in MPN crystals and NPs.^[16]^

To gain insight into the molecular mechanism underlying metal‐phenolic assembly, we investigated the coordination modes of the MPNs at various assembly pHs. The UV‐vis spectra of the MPN particles exhibited the characteristic ligand‐to‐metal charge transfer band, and the coordination states were categorized as Fe‐maltol and Fe‐catechol coordination with mono‐, bis‐, tris‐states by fitting Gaussian peaks to the UV‐vis spectra (Figure S11). Notably, as the assembly pH increased from 4 to 8, the proportion of Fe‐maltol coordination decreased from 97 % to 75 %, whereas the tris‐state of metal‐catechol coordination increased from 2 % to 22 % (Figure 2g). Furthermore, both FTIR spectra of the MPN NPs and crystals featured an Fe−O stretching vibration band at 1269 or 1261 cm^−1^, indicating the coordination network nature of the materials. However, distinct fingerprint regions at 2000–1000 cm^−1^ were observed for the MPN NPs assembled at pH 6, 7, and 8, unlike for the MPN crystals obtained at pH 4 and 5 (Figure 2h). For instance, the C=O stretching at 1665 cm^−1^ of QUE was observed in the spectra of the MPN crystals, whereas the signal shifted to 1647 cm^−1^ for the MPN NPs. Additionally, the C=C aromatic ring stretching signal at 1413 cm^−1^ observed for the NPs was absent in the spectra of the crystals, likely due to the delocalization of electrons on the rings of QUE (Table S2).^[17]^ The stabilizing interactions of the MPN NPs and crystals were further examined using various hydrophobic competitors (e.g., Triton X‐100, Tween 20), π competitors (e.g., dimethylformamide and tetrahydrofuran), an ionic competitor (e.g., NaCl), and a hydrogen bond competitor (e.g., urea) (Figure 2i). MPN NPs readily disassembled after incubation with ethylenediaminetetraacetic acid (EDTA), whereas they remained stable after incubation with other competitor solvents, indicating that coordination is the dominant interaction for NP stabilization. In contrast, MPN crystals disassembled in 100 mM Triton X‐100 and Tween 20 in addition to EDTA, suggesting that both metal coordination and hydrophobic interactions were essential for stabilizing MPN crystals. The presence of hydrophobic interactions can be attributed to the reduced solubility of QUE at lower pH, which induces further assembly of the aggregated metal‐phenolic complexes into crystals.

**Figure 3:**
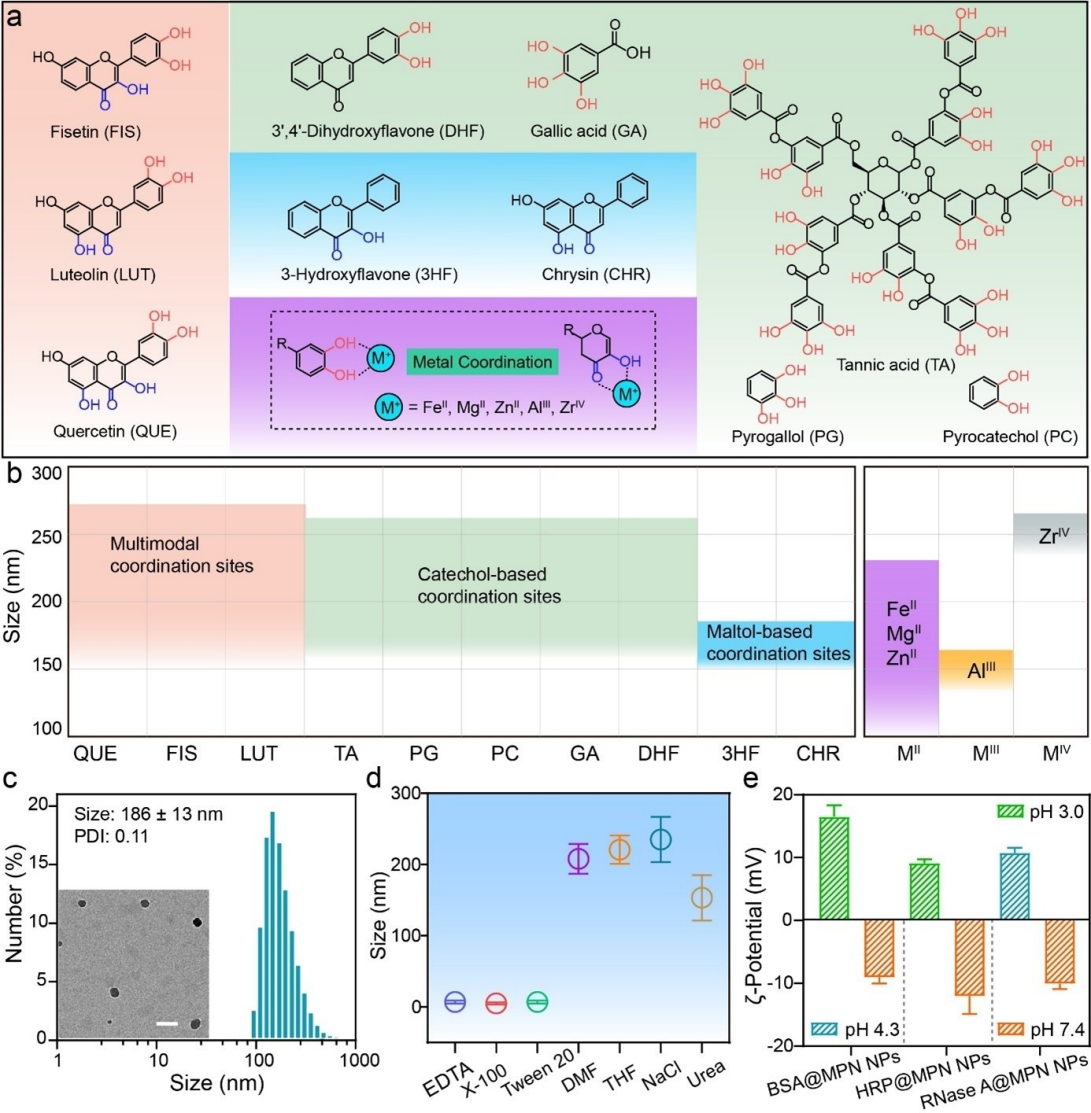
a) Chemical structures of the polyphenols used and their coordination modes with various metal ions (dashed box). Multimodal, catechol‐based, and maltol‐based polyphenol ligands are presented within the orange, green, and blue boxes, respectively. b) Library of MPN NPs with different size ranges fabricated using Fe^II^ and various polyphenols or using QUE and various metal ions in PB (pH 7). c) Size distribution and TEM image (inset) of BSA@MPN NPs. Scale bar is 200 nm. d) Stability of BSA@MPN NPs upon incubation in different media. e) ζ‐Potential values of BSA@MPN NPs, HRP@MPN NPs, and RNase A@MPN NPs at different pH values. Data are shown as the mean±SD (*n*=3). The NP sizes reported in (b–d) and PDI reported in (c) were determined by DLS.

The versatility of this metal‐phenolic assembly strategy was demonstrated with a wide range of phenolic ligands and metal ions to form MPN NPs. Specifically, polyphenols with maltol groups only (e.g., 3‐hydroxyflavone, chrysin), catechol groups only (e.g., tannic acid, gallic acid), and multimodal coordination sites (e.g., fisetin, luteolin) were selected to coordinate with various metal ions with different valences (e.g., Fe^II^, Al^III^, Zr^IV^). A library of MPN NPs with diverse compositions was successfully fabricated to achieve tunable coordination modes and sizes (Figure 3, Figure S12). TEM images and DLS data revealed that the obtained MPN NPs exhibited spherical morphologies and well‐dispersed and adjustable sizes, which may be attributed to the strong electrostatic repulsion caused by their highly negative surface charge (Figures S13 and S14). For instance, the size of MPN NPs assembled using catechol‐based ligands ranged from 160 to 260 nm, whereas NPs produced using maltol‐based ligands were approximately 150–180 nm in size, indicating the stronger metal coordination of the maltol‐based ligands compared to catechol‐based ligands.^[18]^ Additionally, MPN NPs assembled from multimodal coordination ligands exhibited a wider size tunability, ranging from 150 to 270 nm (Figure 3b).

The stability and cytotoxicity of the MPN NPs were examined before exploring their potential for various biomedical applications including biocatalysis, cell targeting, and drug delivery. MPN NPs demonstrated high stability when exposed to various cell culture media (i.e., Dulbecco's phosphate‐buffered saline and Dulbecco's modified Eagle medium with 10 % fetal bovine serum) (Figure S15). However, selective degradation of the MPN NPs under acid conditions (pH≤5) was observed, which is a useful property in potentially achieving controllable drug release within acidic endosomal compartments inside cells.^[19]^ Furthermore, the cytotoxicity of the MPN NPs, assembled from a wide range of compositions, was negligible, even at a particle‐to‐cell ratio of 200 000 : 1 in vitro (Figure S16), highlighting their high biocompatibility.

The loading capability of the MPN NPs with various functional cargos (e.g., proteins and drugs) was assessed. Bovine serum albumin (BSA), a globular protein in blood plasma, was employed as a model protein for the preparation of cargo‐loaded MPN NPs (i.e., BSA@MPN NPs). TEM and AFM images revealed that the spherical BSA@MPN NPs were slightly larger than pristine MPN NPs (Figure 3c, Figures S17 and S18). The assembly mechanism of the BSA@MPN NPs was investigated by incubating the NPs with different competitor solutions. The findings revealed that hydrophobic interactions and coordination bonding played a key role in stabilizing the NPs (Figure 3d). Various functional proteins with different pIs, including insulin (pI 5.3) and cytochrome C (pI 9.6), were then successfully incorporated into the MPN NPs and showed negligible cytotoxicity, even at a particle‐to‐cell ratio of 200 000 : 1 (Figure S19). Furthermore, the protein‐loaded MPN NPs underwent surface charge reversal from negative (at pH 7) to positive (at pH 3 or 4), which is potentially useful for inducing endosomal escape (Figure 3e).^[20]^

**Figure 4:**
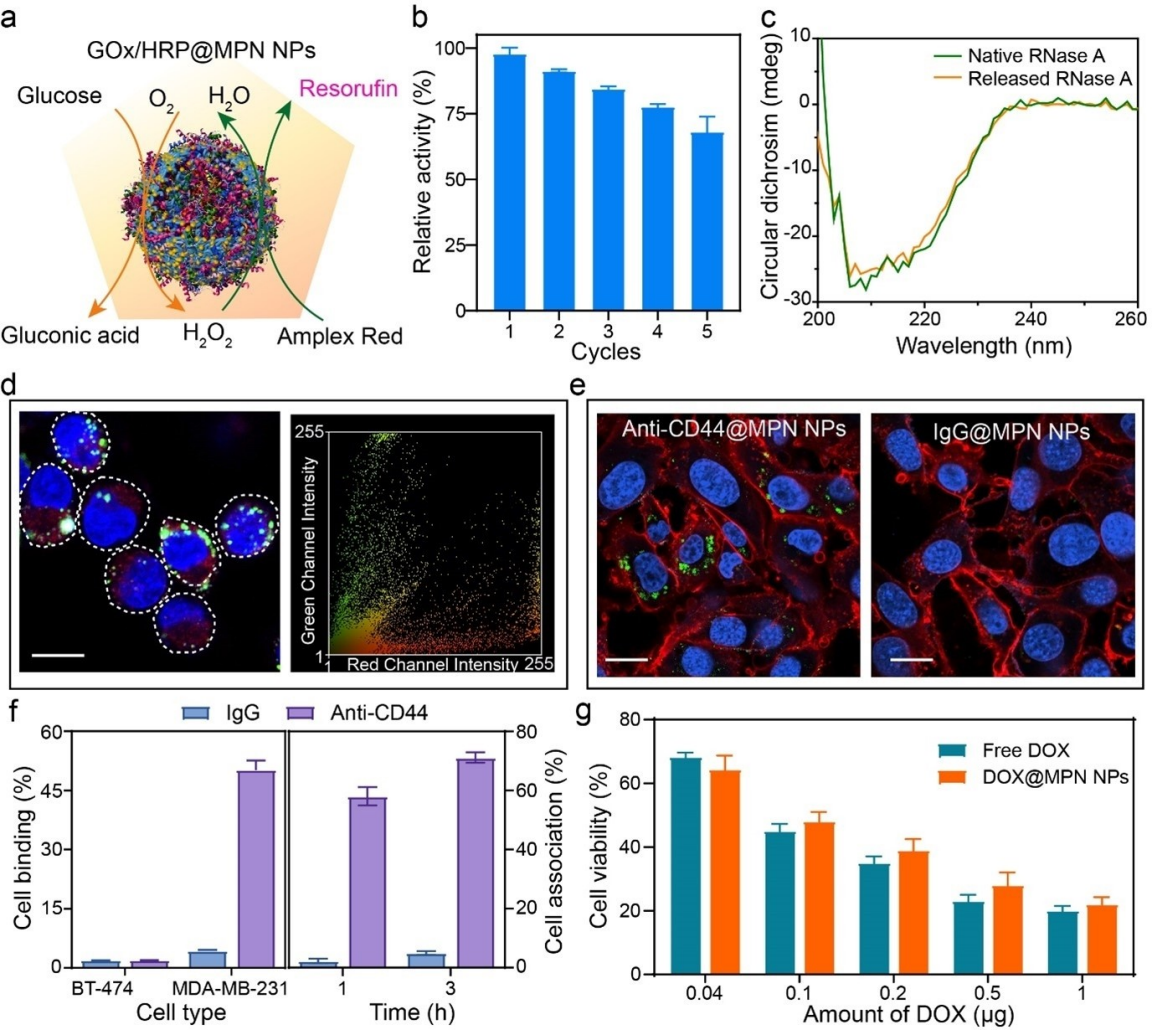
a) Schematic of the cascade reaction by GOx/HRP@MPN NPs, involving glucose oxidation and conversion of amplex red to fluorescent resorufin. b) Relative catalytic activity of HRP@MPN NPs as a function of cycle number. c) CD spectra of native RNase A and RNase A released from NPs. d) CLSM image and related color scatter plot showing the intracellular localization of RNase A@MPN NPs in 3T3 cells after incubation for 4 h. Red, RNase A@MPN NPs; blue, nuclei; green, endosomes and lysosomes. Scale bar is 10 μm. e) CLSM images of anti‐CD44@MPN NPs and IgG@MPN NPs incubated with MDA‐MB‐231 cells for 3 h. Blue, nuclei; red, membrane. Scale bars are 3 μm. f) Flow cytometry analysis of the binding of anti‐CD44@MPN NPs and IgG@MPN NPs to MDA‐MB‐231 or BT‐474 cells after incubation for 1 h at 4 °C (left); percentage of MDA‐MB‐231 cells associated with anti‐CD44@MPN NPs or IgG@MPN NPs after incubation for 1 or 3 h at 37 °C (right). g) Viability of 3T3 cells after incubation with free DOX or DOX@MPN NPs at different drug dosages. Data are shown as the mean±SD (*n*=*5*).

Enzyme‐based proteins (e.g., horseradish peroxidase (HRP), glucose oxidase (GOx)) were incorporated within MPNs to prepare NPs that function as bioreactors (Figure 4a, b). As observed from Figure S20a, HRP@MPN NPs showed comparable catalytic activity to pristine HRP for the oxidation of amplex red in the presence of H_2_O_2_.^[21]^ Notably, HRP@MPN NPs could be effectively recycled at least five times with minimal decreases in activity, unlike pristine HRP, which is challenging to recycle owing to its relatively small size (Figure 4b). Moreover, the MPN NPs could accommodate multiple enzymes, enabling multifunctionality or cascade reactions.^[22]^ For example, GOx within GOx/HRP@MPN NPs catalyzed the conversion of glucose into gluconic acid and produced H_2_O_2_, which was then used by HRP to oxidize amplex red to resorufin (Figure 4a, Figure S20b). Ribonuclease A (RNase A), another model enzyme, can cleave single‐stranded RNA but is prone to denaturation in biological environments if it is not protected (e.g., encased within a carrier). Circular dichroism (CD) spectroscopy demonstrated that incorporating RNase A within MPNs did not alter the conformational structure of the enzyme—the native and released RNase A featured similar CD spectra (Figure 4c). Moreover, the RNase A@MPN NPs exhibited endosomal escape capabilities after incubation with 3T3 cells for 4 h, with a low Pearson's correlation coefficient value of 0.45, owing to their charge reversal properties under acid conditions (Figure 4d, Figure S21).

Therapeutic molecules were also integrated with MPNs to produce NPs with cell‐targeting properties. Anti‐CD44 antibodies can specifically target CD44 overexpressing cells (e.g., MDA‐MB‐231 cells) while exhibiting limited interactions with CD44 minimal expressing cells (e.g., BT‐474 cells).^[23]^ The cell binding and targeting ability of anti‐CD44@MPN NPs were characterized by confocal laser scanning microscopy (CLSM) and flow cytometry (Figure 4e, Figure S22). Specifically, the anti‐CD44@MPN NPs exhibited higher binding specificity to MDA‐MB‐231 cells (i.e., ≈70 % cell association after 3 h) than the negative control, i.e., immunoglobulin G (IgG)@MPN NPs (i.e., ≈5 % cell association after 3 h) (Figure 4f, Figure S23). This suggests that the inherent targeting ability of the antibodies was retained after co‐assembly with the MPNs. Additionally, small therapeutic molecules (e.g., doxorubicin (DOX)) were incorporated during NP fabrication. The DOX@MPN NPs exhibited comparable anticancer activity to that of free DOX at a given free DOX dosage (Figure 4g). Moreover, the release rates of DOX from DOX@MPN NPs could be controlled by adjusting the pH conditions, with rapid release observed under acidic conditions (Figure S24).

## Conclusion

We have reported a simple approach for the direct assembly of MPN NPs at ambient temperature in the absence of seeding agents or templates. The type of buffer (e.g., phosphate buffer) was found to influence NP formation, and the sizes and morphologies of the MPN NPs were engineered by adjusting the reaction time, precursor concentration, and coordination mode at different pH values. The versatility of the NP assembly process was demonstrated by the suite of MPN NPs fabricated using a wide range of polyphenols and metal ions. Moreover, various functional components (e.g., enzyme, drug) were incorporated to create functional NPs for biocatalysis, cell targeting, and drug delivery studies. This study provides valuable insight into the coordination modes of metal‐organic complexes and introduces a simple, rapid, and robust method for the direct assembly of metal‐organic NPs. Considering the negligible cytotoxicity and innate functionalities of natural polyphenols (e.g., antibacterial, anti‐inflammatory, and anticancer effects), the library of MPN NPs reported herein holds potential for various biomedical applications. Furthermore, this work is expected to provide a framework for designing a wide array of nonpolyphenol‐based metal‐organic NPs with potential in catalysis, environmental, and agricultural applications.

## Conflict of interest

The authors declare no conflict of interest.

1

## Supporting information

As a service to our authors and readers, this journal provides supporting information supplied by the authors. Such materials are peer reviewed and may be re‐organized for online delivery, but are not copy‐edited or typeset. Technical support issues arising from supporting information (other than missing files) should be addressed to the authors.

Supporting Information

## Data Availability

The data that support the findings of this study are available from the corresponding author upon reasonable request.
