# Supplementary material for: Direct Assembly of Metal‐Phenolic Network Nanoparticles for Biomedical Applications
Source: Angew Chem Int Ed Engl. 2023 Oct 6;62(45):e202312925. doi: 10.1002/anie.202312925 (PMC10953434; doi:10.1002/anie.202312925)
Supplement: Supplementary file 1 — Supporting Information [file ANIE-62-0-s001.pdf]

Angewandte Chemie
Eine Zeitschrift der Gesellschaft Deutscher Chemiker

Supporting Information

Direct Assembly of Metal-Phenolic Network Nanoparticles for Biomedical Applications

*W. Xu, Z. Lin, S. Pan, J. Chen, T. Wang, C. Cortez-Jugo, F. Caruso**

Section S1. Experimental

Materials

Quercetin (QUE), luteolin (LUT), fisetin (FIS), 3-hydroxyflavone (3HF), chrysin (CHR), 3′,4′-dihydroxyflavone (DHF), tannic acid (TA), gallic acid (GA), pyrogallol (PG), pyrocatechol (PC), iron(II) chloride tetrahydrate ($FeCl_2·4H_2O$), zinc nitrate hexahydrate ($Zn(NO_3)_2·6H_2O$), aluminum chloride hexahydrate ($AlCl_3·6H_2O$), magnesium chloride hexahydrate ($MgCl_2·6H_2O$), zirconium(IV) chloride ($ZrCl_4$), sodium phosphate dibasic heptahydrate, sodium phosphate monobasic monohydrate, sodium hydroxide, hydrogen chloride, (3-(N-morpholino)propanesulfonic acid (MOPS), 2-(N-morpholino)ethanesulfonic acid (MES), bicine, citrate, bis-tris, sodium phosphate dibasic heptahydrate, sodium phosphate monobasic monohydrate, fluorescein isothiocyanate (FITC), rhodamine B (RhB), glucose, urea, Triton X-100, Tween 20, Dulbecco's modified Eagle medium (DMEM), Dulbecco's phosphate-buffered saline (DPBS), and fetal bovine serum (FBS) were purchased from Sigma-Aldrich (USA). Dimethyl sulfoxide (DMSO), dimethylformamide (DMF), tetrahydrofuran (THF), 1,4-dioxane, methanol, and ethanol were purchased from Chem-Supply. Insulin (INS), cytochrome C (CYC), immunoglobulin G (IgG), anti-CD44, bovine serum albumin (BSA), horseradish peroxidase (HRP), glucose oxidase (GOx), amplex red, and ribonuclease A (RNase A) were purchased from Thermo Fisher Scientific (USA). Doxorubicin (DOX) and 2,3-bis[2-methoxy-4-nitro-5-sulfophenyl]-2*H*-tetrazolium-5-carboxyanilide inner salt (XTT) were obtained from Life Technologies. Milli-Q water with a resistivity of 18.2 MΩ cm was obtained from a three-stage Millipore Milli-Q plus 185 purification system (Millipore Corporation, USA).

Characterization

UV–vis absorption spectra were recorded on a Specord 250 Plus spectrophotometer (Analytik Jena AG). Fourier transform infrared (FTIR) spectroscopy analysis was conducted on a Tensor II FTIR spectrometer. Scanning electron microscopy images were obtained using an FEI Teneo VolumeScope with an operating voltage of 10 kV. Atomic force microscopy (AFM) experiments were conducted using a JPK NanoWizard II BioAFM instrument. Confocal laser scanning microscopy (CLSM) images were taken with a Nikon A1R+ laser scanning confocal microscope (Nikon Corporation, Japan). The number size distribution and ζ-potentials of particles were measured via dynamic light scattering (DLS) on a Zetasizer Nano ZS instrument (Malvern Instrument, UK). Small-angle X-ray scattering (SAXS) and X-ray absorption spectroscopy (XAS) data were collected at the SAXS and XAS beamlines of the Australian Synchrotron facility, respectively, part of the Australian Nuclear Science and Technology Organisation (ANSTO). Samples were examined using the small-/wide-angle X-ray scattering beamline (16 keV, 7000 mm camera length using Pilatus 1M and 200K detectors, transmission mode). Scatterbrain software was used for the analysis. X-ray powder diffraction was performed on a Bruker D8 Advance diffractometer. Step size was set as 0.02. Super-resolution microscopy images were acquired on a Zeiss Elyra 7 lattice structured illumination microscopy system with a 2× PCO.EDGE 4.2 CLHS sCMOS camera. A 488 nm laser was used for the excitation of the FITC-labeled nanoparticles (NPs). The metal content in the NPs was determined by inductively coupled plasma optical emission spectrometry (ICP-OES) on an ICP Varian 710-ES instrument.

Fabrication of Metal–Phenolic Network Nanoparticles (MPN NPs)

All polyphenol and metal solutions were prepared freshly for immediate use. The standard protocol used for MPN NP preparation was as follows: 1000 µL of 10 mM phosphate buffer (PB) was added to a vial and stirred at 1100 rpm. To the vial, 80 µL of $FeCl_2·4H_2O$ (10 mg mL^{-1} in water) and 30 µL of QUE (5 mg mL^{-1} in methanol) were added successively and stirred for 30 min at room temperature. The assembled MPN NPs were washed three times by centrifugation (9000 *g*, 5 min) to remove excess materials. The resultant MPN NPs were dispersed in either Milli-Q water or buffer solution for future use. When examining the effect of assembly pH in the range of 4–8, the pH was adjusted accordingly by adding 1 mM NaOH or 1 mM HCl into pH 7 PB buffer.

For the fabrication of MPN NPs using other metal ions (i.e., Mg^{II}, Zn^{II}, Al^{III}, Zr^{IV}) and polyphenols (i.e., CHR, 3HF, DHF, PC, PG, TA, GA, FIS, LUT), a metal-to-polyphenol molar ratio of 8:1 was used and the same fabrication process was applied.

Coordination of MPN Particles

UV–vis spectrophotometry was used to determine the coordination sites of the MPN particles. The samples were prepared as follows: the pellets of MPN particles assembled at different pHs were washed three times with water and then redispersed in water for the measurements. FTIR spectroscopy was used to determine the coordination between Fe^{II} and QUE in the solid state. The corresponding MPNs

fabricated at different pHs were collected and freeze-dried for the FTIR spectroscopy measurements. The Gaussian function fitting approach was used to perform curve fitting.

Stability of MPN Particles
To evaluate the stability of the MPN NPs under different conditions, MPN NPs were dispersed in 100 mM glycine–HCl (pH 3.0), 100 mM sodium acetate (pH 5.0), 100 mM sodium acetate (pH 6.5), 100 mM PBS (pH 7.4), Milli-Q water, DPBS, or DMEM with 10% FBS for the desired time. To determine the possible driving force for the assembly of MPN NPs and MPN crystals, the pellets were incubated in 100 mM of urea, Tween 20, Triton X-100, ethylenediaminetetraacetic acid (EDTA), DMF, or THF for 15 h. Changes in size were measured on a Zetasizer Nano-ZS instrument. Data are shown as the mean ± standard deviation (SD) of three independent measurements.

Quantification of Iron and QUE in Fe^{II}–QUE MPN Particles
The content of iron and QUE in Fe^{II}–QUE MPN particles assembled at different pH was measured by ICP-OES and UV–vis spectrophotometry. First, an iron ICP standard solution was diluted to 0.1, 0.5, 1, 5, and 10 ppm with 5% nitric acid (HNO_3) to construct calibration curves. To determine the content of iron, Fe^{II}–QUE MPN particles were disassembled in 65% HNO_3 and then diluted with Milli-Q water to 5% HNO_3 for ICP analysis. To quantify the amount of QUE, a calibration curve of QUE was constructed, and the disassembled Fe^{II}–QUE MPN particles were analyzed by UV–vis spectrophotometry at an absorption wavelength of 371 nm.

Fabrication and Characterization of Drug-/Protein-Loaded MPN NPs
The assembled MPN NPs were complexed with cargo proteins (i.e., BSA, CYC, HRP, RNase A, GOx, INS, IgG, anti-CD44) or small molecule drug (i.e., DOX) at different weight ratios in deionized water or buffer for 30 min. Size and ζ-potential of the prepared NPs were characterized by Zetasizer Nano ZS. Proteins with different isoelectric points (pIs), such as BSA (4.6), HRP (7.2), and RNase A (9.6), were loaded into MPN NPs and the ζ-potentials of the protein@MPN NPs were measured under different pH buffers. The stability of BSA@MPN NPs under different conditions was assessed by incubation in 100 mM of urea, Tween 20, Triton X-100, EDTA, NaCl, DMF, or THF for 15 h.

Cell Viability by XTT Assay
XTT-based in vitro cytotoxicity assay was performed to assess the cell toxicity of the MPN NPs, protein@MPN NPs, and DOX@MPN NPs. XTT was dissolved in complete DMEM (with 10% FBS) to prepare a 0.2 mg mL^{-1} solution, and phenazine methosulfate (PMS) was dissolved in DPBS to prepare a 1 mM solution. The XTT reagent was activated by mixing with PMS solution at a volume ratio of 400:1. 3T3 cells were seeded on a 96-well plate at a cell density of 2×10^4 cells per well. To determine the cell viability of MPN NPs and protein@MPN NPs, the NP-to-cell ratio was set to 200000:1. To determine the cell viability of DOX@MPN NPs, the cells were incubated with different dosages of DOX loaded in the DOX@MPN NPs and with free DOX as control. After incubation for 24 h at 37 °C, the media in the 96-well plate was aspirated and replaced with 100 µL of fresh activated XTT media. The cells were further incubated for 4 h at 37 °C and the absorbance at 475 nm was measured relative to nontreated cells.

Catalytic Activity Test
The activity of HRP loaded in MPN NPs was evaluated by the H_2O_2–amplex red colorimetric reaction. Briefly, MPN NPs, free HRP, or HRP@MPN NPs were mixed with 10 mM PBS solution (pH 7.4) containing 20 mM H_2O_2 and 1 mg mL^{-1} amplex red. Changes in the absorbance of the red oxidation product (resorufin) at 560 nm were monitored by UV–vis spectroscopy. The kinetic behavior of HRP was studied by monitoring the absorbance at 560 nm at 10 s intervals by UV–vis spectroscopy.

Recycling experiments with HRP@MPN NPs were performed under the same condition as described above. After one cycle, HRP@MPN NPs were retrieved by centrifugation (9000 *g*, 5 min), washed with PBS, and reused in the subsequent cycle of catalysis.

For the cascade reaction involving GOx/HRP@MPN NPs, glucose (10 mg mL^{-1}) was used to initiate the two tandem reactions in 10 mM PBS (pH 7.4) and the total volume of the reaction solution was 200 µL. After completion of the reaction (20 min at 37 °C), the UV–vis absorption spectra were recorded.

Conformation Analysis of RNase A
The secondary structure of RNase A released from MPN NPs was determined by circular dichroism (CD) spectroscopy. The native RNase A was dissolved in DPBS solution, at the final concentration of 0.4 mg mL^{-1}. CD measurements were performed on a Jasco J-810 CD spectropolarimeter at 25 °C with a cell length of 0.1 cm. All samples were scanned from 190 to 260 nm and replicated three times at a

resolution of 1.0 nm and scanning speed of 100 nm min^{-1}. All CD data were expressed as mean residue ellipticity.

Endosomal Escape of RNase A@MPN NPs
3T3 cells were seeded into 8-well Lab-Tek chambered cover glass slides at a cell density of 4 × 10^4 cells per well and then cultured in 400 µL of DMEM supplied with 10% FBS for 24 h to allow cellular adhesion on substrates. Then, RhB-labeled RNase A@MPN NPs were added to the cells and incubated for 4 h at 37 °C. After incubation, the treated cells were gently washed three times with DPBS and stained with LysoTracker Green for endosome/lysosome labeling. For nucleus staining, cells were gently washed three times with DPBS and incubated with Hoechst 33342 (1 µg mL^{-1}) for 10 min. Live cell imaging was performed by CLSM using a 40× water immersion objective. Pearson's correlation coefficient (PCC) and color scatter plots were obtained from WCIF ImageJ software. The experiments were repeated in triplicates, and five representative cell images (>50 cells) were used to calculate the PCC values.

Cell Targeting Studies of Antibody@MPN NPs via Flow Cytometry
MDA-MB-231 cells and BT-474 cells were seeded in a 24-well plate at a cell density of 1 × 10^5 cells per well and then cultured in DMEM supplied with 10% FBS at 37 °C for 14 h. Following this, FITC-labeled anti-CD44@MPN NPs and IgG@MPN NPs were added to the cells at a particle-to-cell ratio of 20000:1 and then incubated for 1 h at 4 °C for the cell binding study or 1 and 3 h at 37 °C for the cell association study. After incubation, the cells were gently washed three times with DPBS, dissociated using trypsin solution, and analyzed on an Apogee A50-Micro flow cytometer (Apogee Flow Systems, UK). The degree of cell binding or association of the NPs was evaluated by using the percentage of cells that exhibited stronger fluorescence intensity than the control untreated cells.

Cell Targeting Studies of Antibody@MPN NPs via Confocal Microscopy
MDA-MB-231 cells and BT-474 cells were seeded in 8-well Lab-Tek chamber slides at a cell density of 4 × 10^4 cells per well and then cultured in DMEM supplemented with 10% FBS at 37 °C for 14 h. Then, FITC-labeled anti-CD44@MPN NPs and IgG@MPN NPs were added to the cells at a particle-to-cell ratio of 20000:1 and then incubated for 3 h at 37 °C. After incubation, the cells were gently washed three times with DPBS, fixed with paraformaldehyde (4% in DPBS) for 15 min at 37 °C, then stained with wheat germ agglutin-594 (5 µg mL^{-1}) at room temperature for 15 min, followed by Hoechst 33342 (2 µg mL^{-1}) staining at room temperature for 10 min. Finally, cells were imaged and captured by CLSM.

pH-Responsive Release of DOX
A standard curve of DOX was prepared by measuring the absorption wavelength at 480 nm of DOX solutions with different known concentrations. The loading amount of DOX in NPs was measured using the standard curve by UV–vis spectrophotometry. To determine the cumulative release of DOX under different pH values, the DOX@MPN NPs were resuspended in 200 µL of buffer solutions of different pHs (7.4, 6.0, or 5.0). At desired time points, the NPs were centrifugated and the supernatant was collected for DOX concentration quantification.

Minimum Information Reporting in Bio–Nano Experimental Literature (MIRIBEL)
The studies conducted herein, including material characterization, biological characterization, and experimental details, conform to the MIRIBEL reporting standard for bio–nano research,[1] and we include a companion checklist of these components herein.

Section S2. Supporting Figures

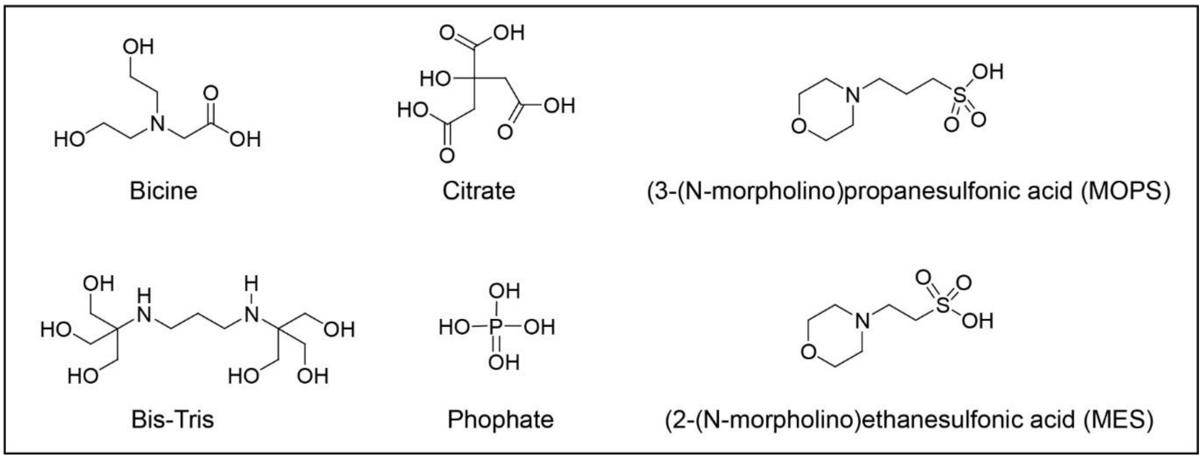

Figure S1. Chemical structures of buffering agents that were investigated in this study.

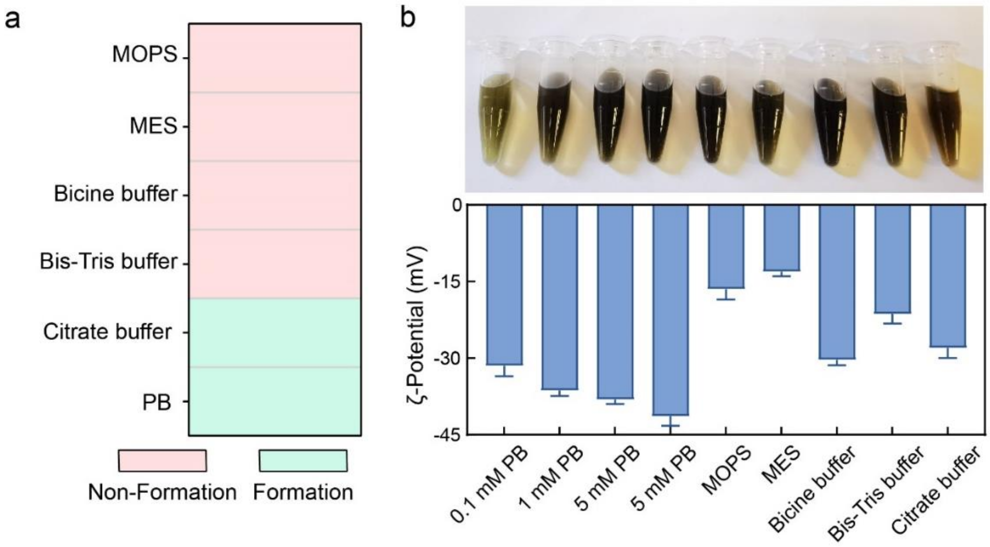

Figure S2. (a) Summary of the formation of MPN NPs using buffering agents. (b) Photograph and ζ-potential values of MPN NPs fabricated using different buffering agents. Data are shown as the mean ± SD (n = 3). The buffer solutions featured a comparable pH (i.e., pH 7).

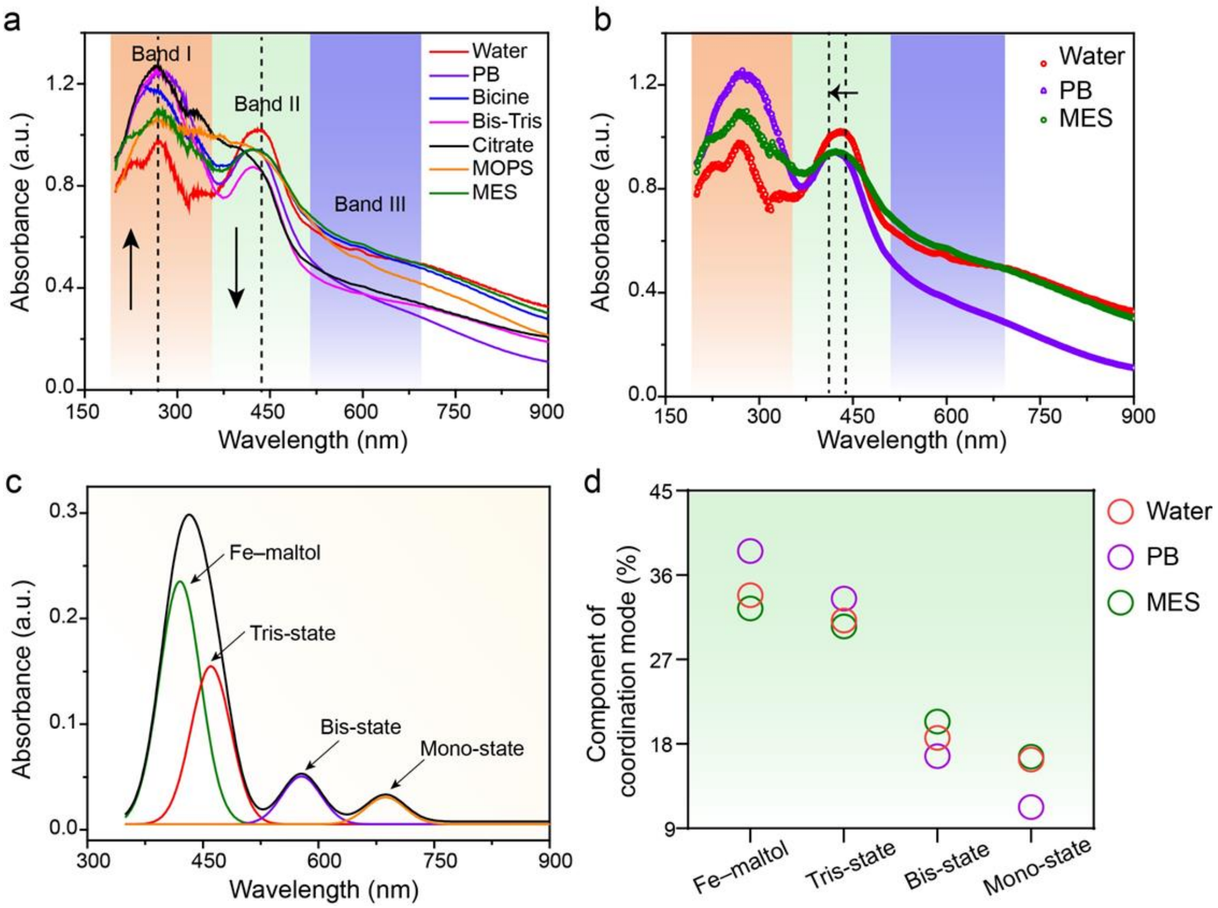

Figure S3. (a) UV–vis spectra of MPNs prepared using different buffering agents and water. Band I was assigned to π–π* transition in QUE (the arrow indicates increases in the absorbance with the use of buffering agents relative to water). Band II was attributed to Fe–maltol coordination (the arrow indicates decreases in the absorbance with the use of buffering agents relative to water). Band III was attributed to Fe–catechol coordination. (b) UV–vis spectra of MPNs prepared in water, PB, or MES. Maximum absorption of Band II shifted from 430 nm (water) to 418 nm (PB and MES). (c) Peak fitting of MPNs prepared in PB with the black line representing the cumulative measurement. The fitted peaks correspond to the Fe–maltol coordination, and tri-state, bis-state, and mono-state Fe–catechol coordination. (d) Percentage of Fe–maltol, and mono-, bis-, and tris-state Fe–catechol coordination states, calculated from the area under the curve of Band II and Band III for MPNs prepared in water, PB, and MES.

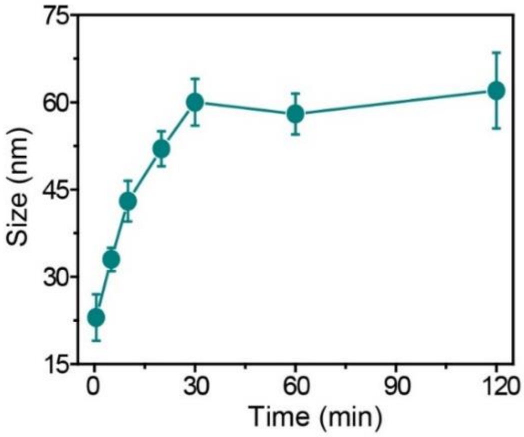

Figure S4. Size of MPN NPs (prepared using PB), as determined by DLS, as a function of time during the assembly process. Note that 1 mg mL^{-1} Fe^{II} and 0.5 mg mL^{-1} QUE were used. Data are presented as the mean ± SD (n = 3).

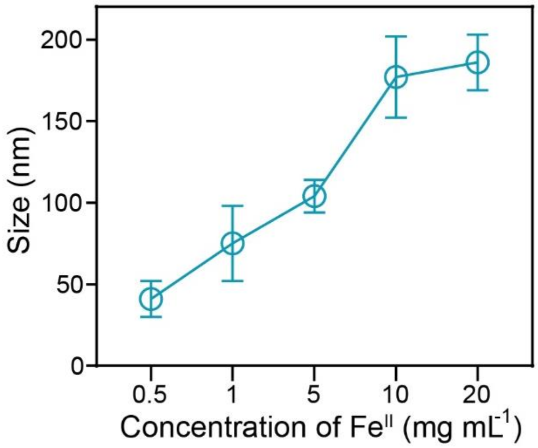

Figure S5. Size of MPN NPs (prepared using PB), as determined by DLS, as a function of concentration of Fe^{II}. Note that the concentration of QUE was also varied, while the molar ratio of Fe^{II}-to-QUE was maintained at 8:1. Data are presented as the mean ± SD (n = 3).

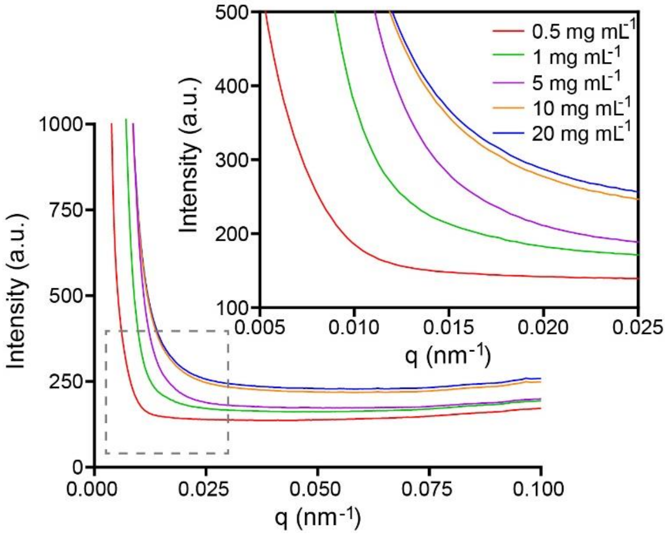

Figure S6. SAXS data of MPN NPs assembled at different concentrations of Fe^{II}. The molar ratio of Fe^{II}-to-QUE was maintained at 8:1.

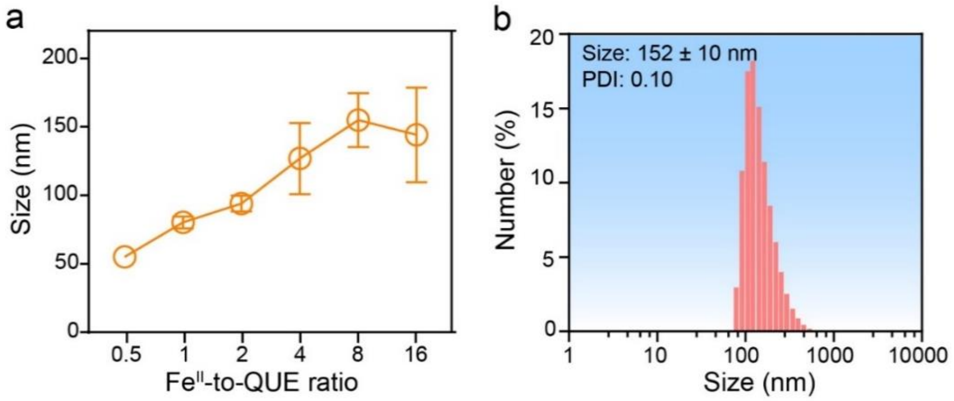

Figure S7. (a) Size of MPN NPs, as determined by DLS, as a function of Fe^{II}-to-QUE molar ratio. (b) Number size distribution and polydispersity index (PDI) of the MPN NPs. The MPN NPs were fabricated by sequentially adding 80 µL of Fe^{II} (10 mg mL^{-1}) and 30 µL of QUE (5 mg mL^{-1}) to a vial containing 1 mL of PB buffer (10 mM).

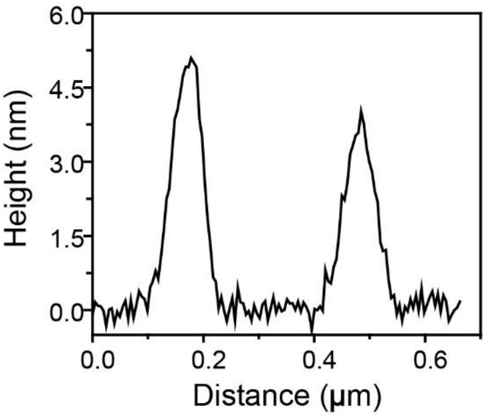

Figure S8. Representative height profile of MPN NPs measured by AFM.

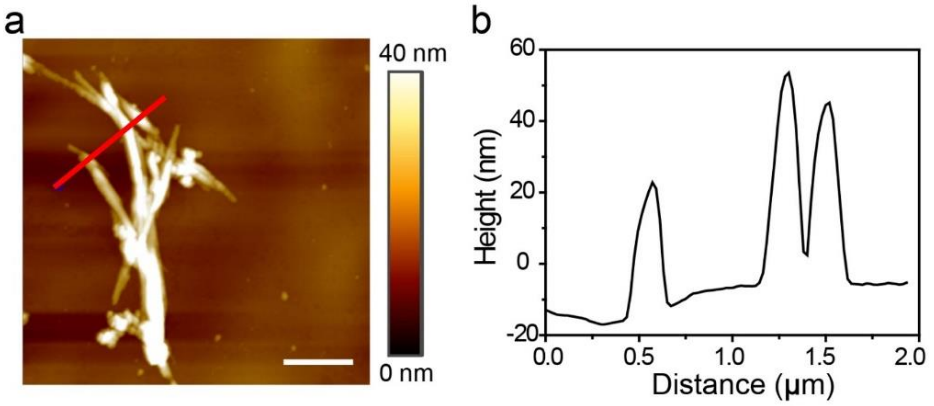

Figure S9. (a) AFM image and (b) corresponding thickness profile of MPN crystals prepared at pH 4. Scale bar is 1000 nm.

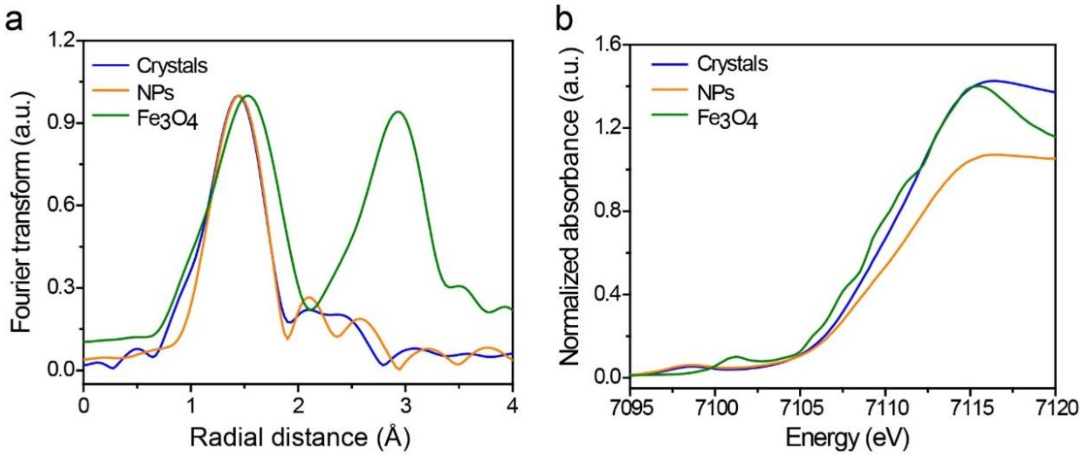

Figure S10. (a) Fe K-edge extended X-ray absorption fine structure (EXAFS) spectra and (b) X-ray absorption near-edge structure (XANES) spectra of MPN crystals, MPN NPs, and Fe_3O_4 reference compound.

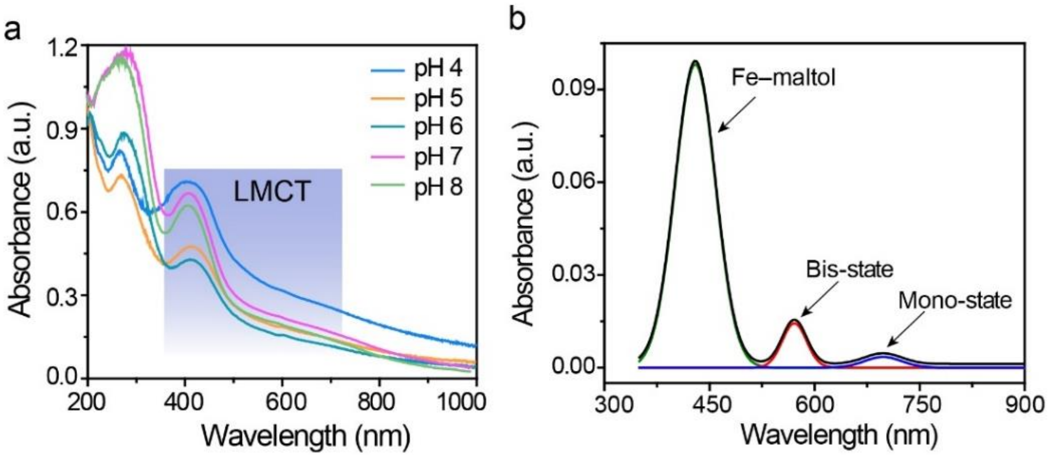

Figure S11. (a) UV–vis spectra of MPN particles prepared at different assembly pH values. (b) Peak fitting of MPN NPs prepared at pH 6. The fitted peaks correspond to Fe–maltol coordination, and mono-, and bis-state Fe–catechol coordination.

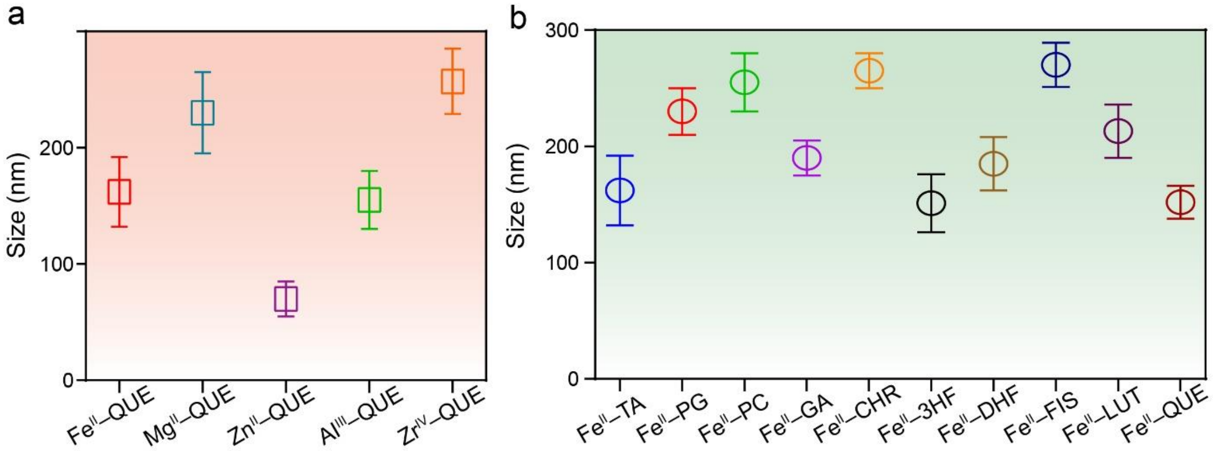

Figure S12. Size of MPN NPs prepared using (a) QUE and various metal ions and (b) Fe^{II} and various polyphenols. The NP size was determined by DLS. Data are presented as the mean ± SD, $n = 3$.

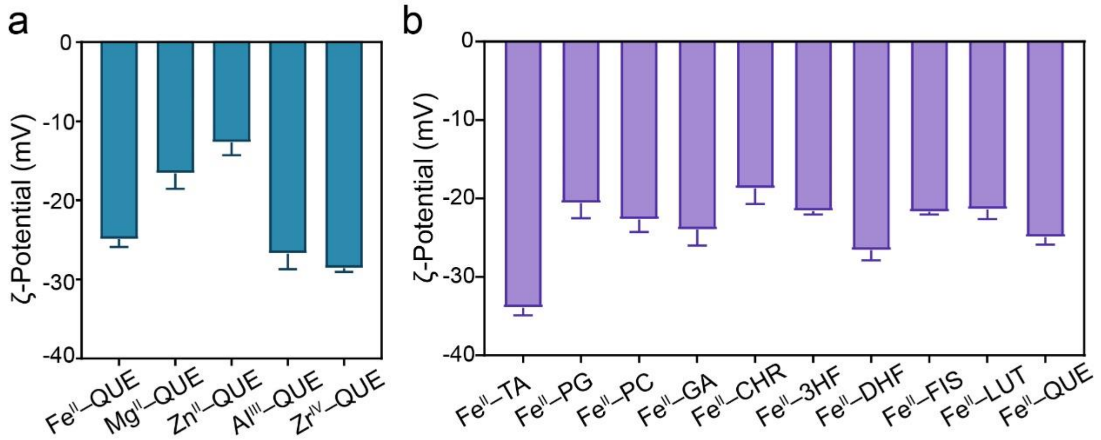

Figure S13. ζ-Potential values of MPN NPs prepared using (a) QUE and various metal ions and (b) Fe^{II} and various polyphenols. Data are presented as the mean ± SD, n = 3.

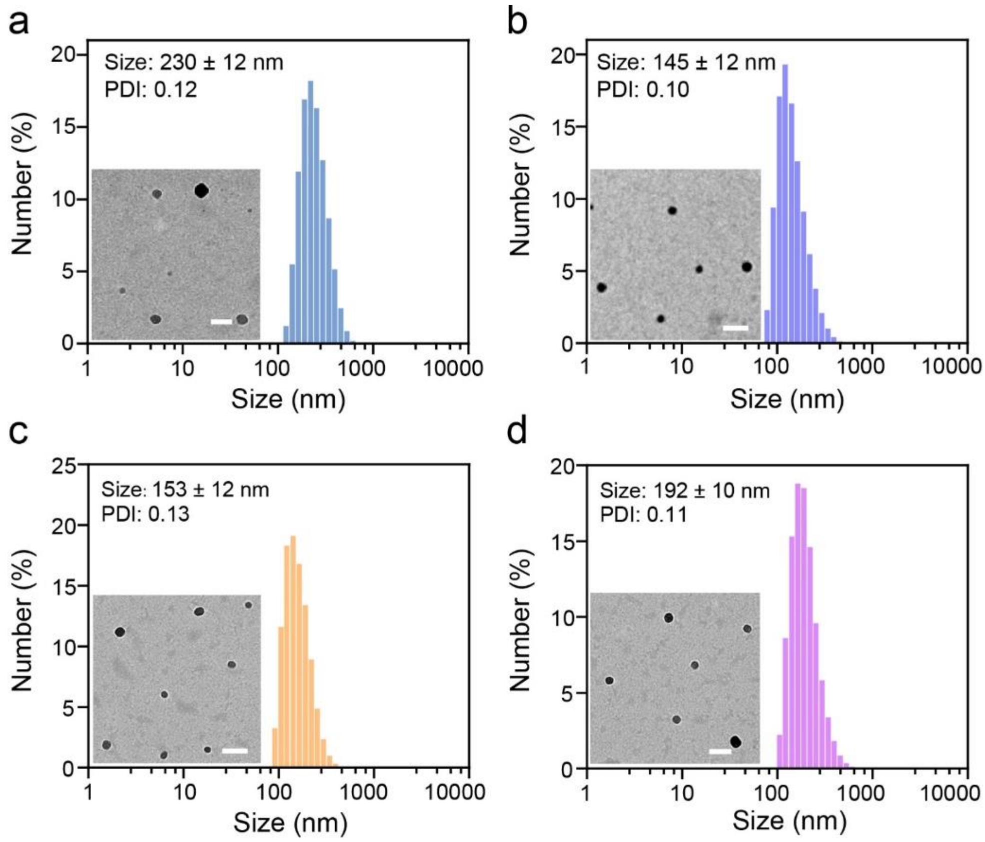

Figure S14. DLS data and corresponding TEM images of representative MPN NPs prepared using various metal ions and polyphenols, including (a) Mg^{II}–QUE NPs, (b) Al^{III}–QUE NPs, (c) Fe^{II}–TA NPs, and (d) Fe^{II}–GA NPs. The size and PDI of the NPs were determined by DLS. Scale bars are 200 nm.

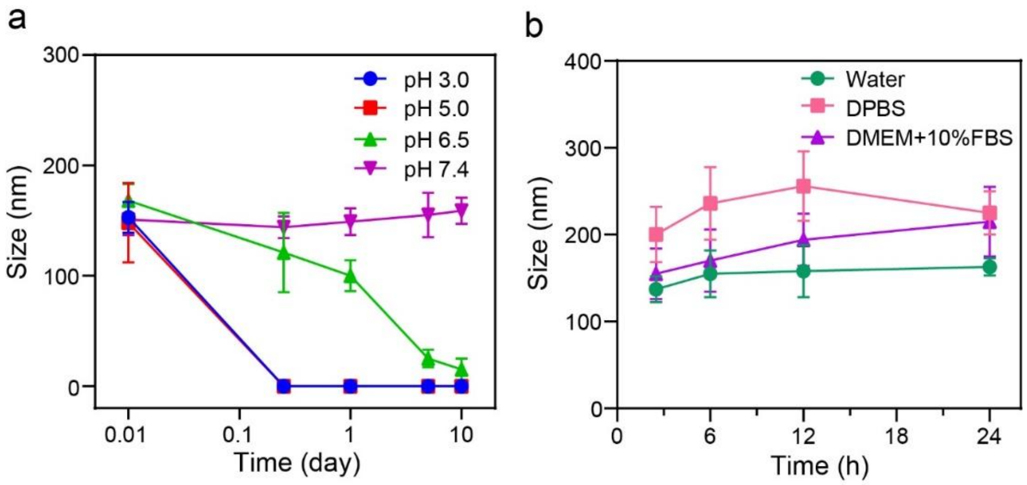

Figure S15. Stability of MPN NPs upon incubation in (a) different pH solutions and (b) different culture media over time. The size of the NPs was determined by DLS. Data are presented as the mean ± SD, *n* = 3.

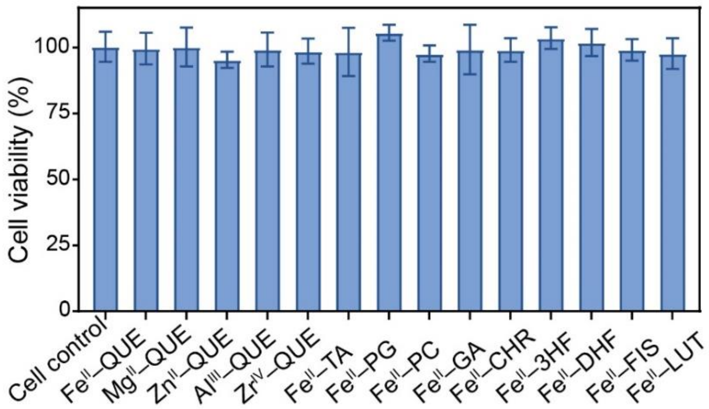

Figure S16. Viability of 3T3 cells after incubating with MPN NPs fabricated from different metal ions and polyphenols at a particle-to-cell ratio of 200000:1. The particle concentration is 4×10^9 particles per mL.

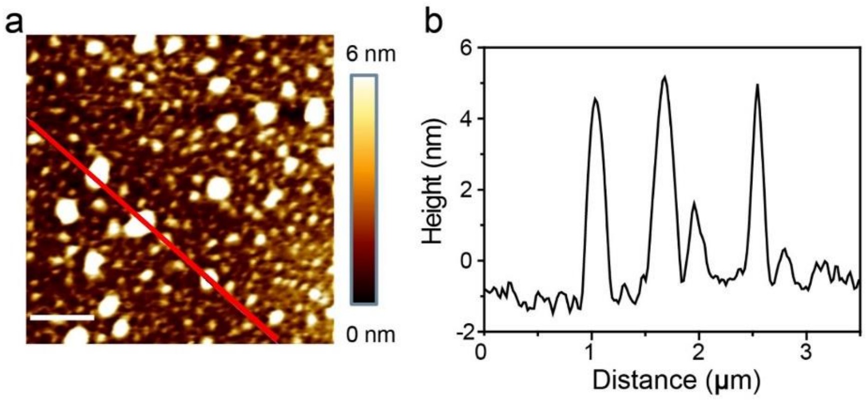

Figure S17. (a) AFM image and (b) corresponding thickness profile of BSA@MPN NPs. Scale bar is 200 nm.

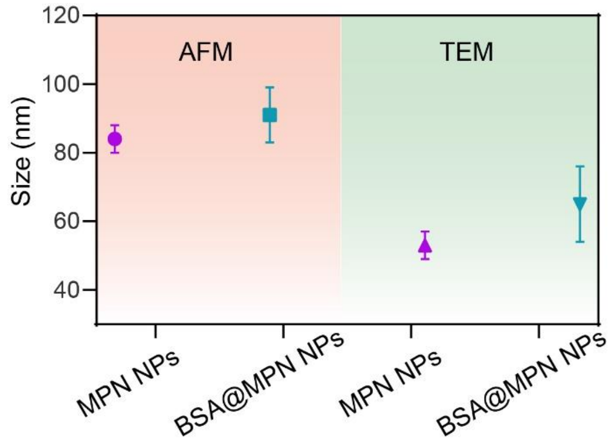

Figure S18. Size of MPN NPs and BSA@MPN NPs determined by TEM and AFM. Data are shown as the mean ± standard deviation (n = 15).

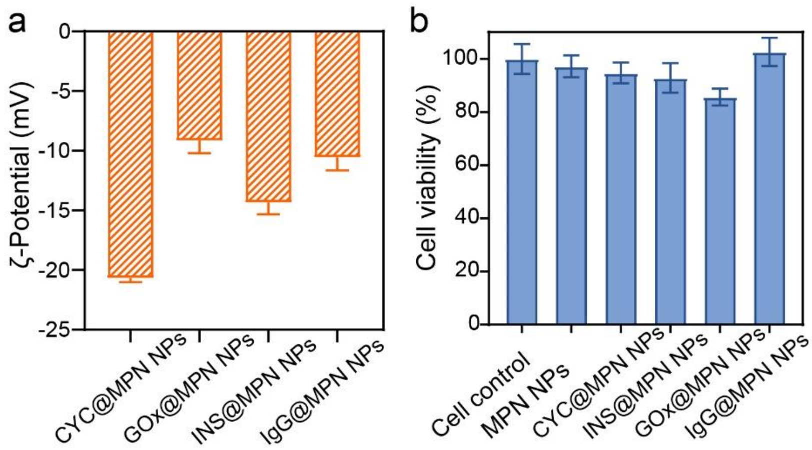

Figure S19. (a) ζ-Potential values of protein@MPN NPs measured at pH 7. (b) Viability of 3T3 cells after incubating with different protein@MPN NPs at a particle-to-cell ratio of 200000:1. The particle concentration is 4 × 10^9 particles per mL. Data are presented as the mean ± SD, n = 3.

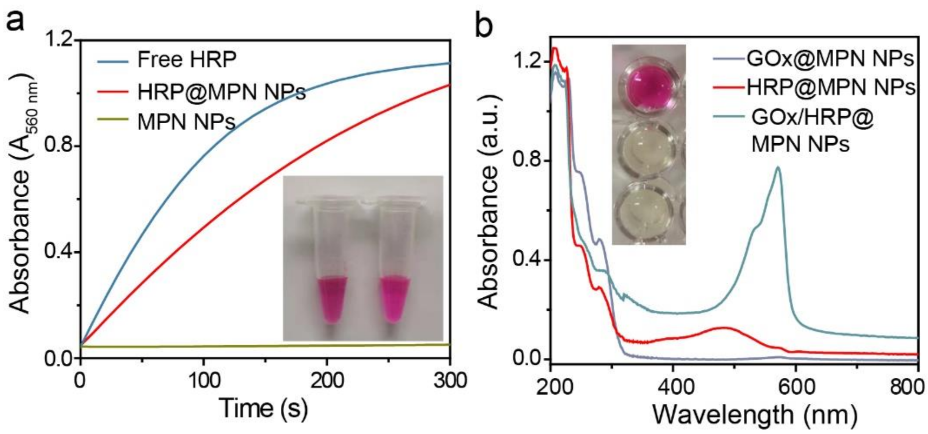

Figure S20. (a) Time-dependent absorbance changes upon oxidation of amplex red by different catalytic systems: free HRP, MPN NPs, and HRP@MPN NPs. (b) UV–vis spectra of the cascade reaction using different single- and multicomponent NPs. Inset: color changes using the different NP systems.

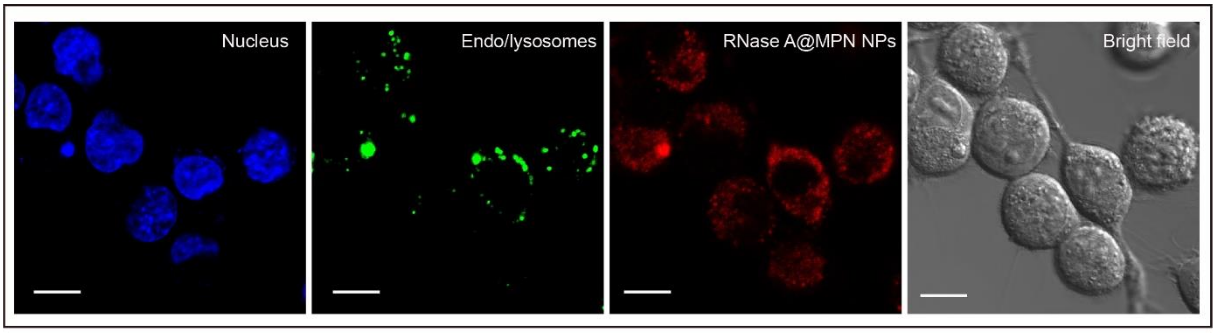

Figure S21. CLSM images of 3T3 cells incubated with RNase A@MPN NPs for 4 h at a particle-to-cell ratio of 20000:1. Red, RNase A@MPN NPs; green, endosomes and lysosomes; blue, nuclei. Scale bars are 10 μm.

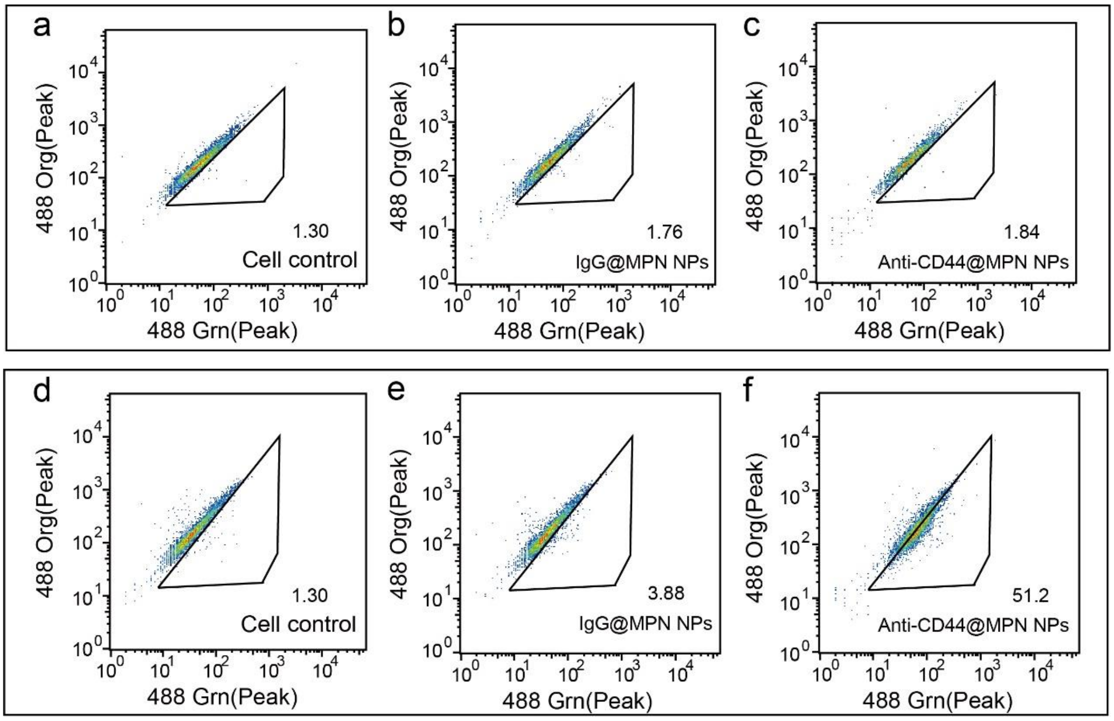

Figure S22. Flow cytometry data showing cell binding of anti-CD44@MPN NPs or IgG@MPN NPs to (a–c) BT-474 cells or (d–f) MDA-MB-231 cells after incubation at 4 °C for 1 h.

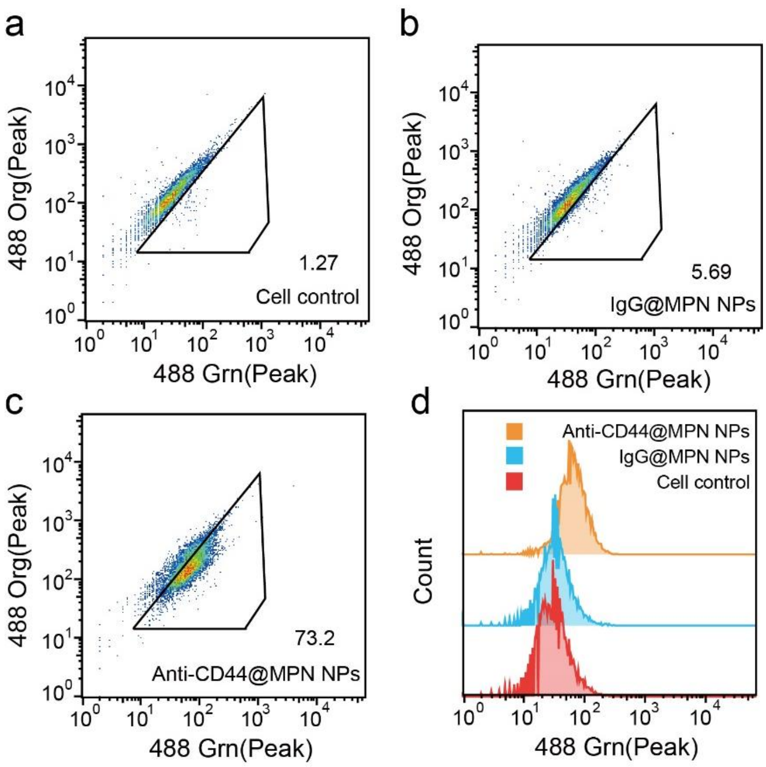

Figure S23. Flow cytometry data showing cell association of anti-CD44@MPN NPs or IgG@MPN NPs with MDA-MB-231 cells after incubation at 37 °C for 3 h.

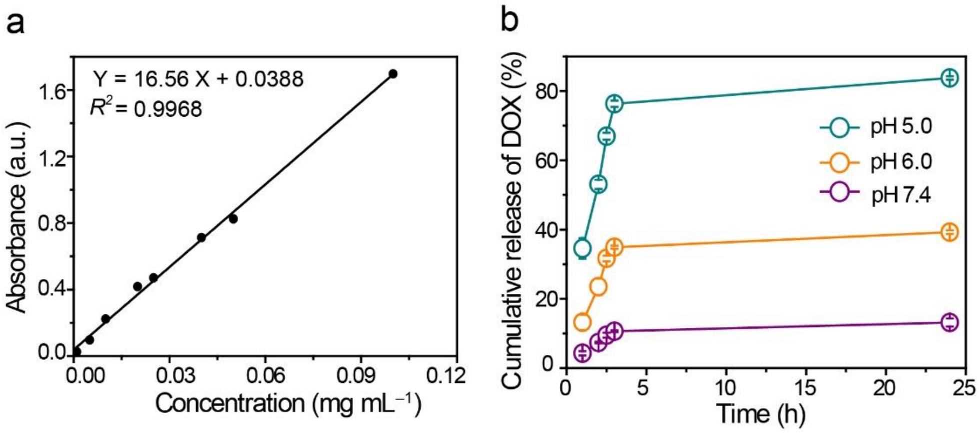

Figure S24. (a) Standard curve of DOX concentration at 480 nm as measured by UV–vis spectrometry. (b) In vitro release profiles of DOX from DOX@MPN NPs under different pH conditions. Data are presented as the mean ± SD, n = 3.

Section S3. Supporting Tables

Table S1. Size and PDI of MPN NPs fabricated using different concentrations of PB

Concentration of PB (mM)	Size (nm)	PDI
0.1	N/A	N/A
1	318 ± 36	0.22
5	231 ± 13	0.15
10	177 ± 24	0.11

NA, not applicable. The size and PDI of the NPs were determined by DLS.

Table S2. FTIR characteristic bands of Fe^{II}–QUE crystals (obtained at pH 4–5) and Fe^{II}–QUE NPs (obtained at pH 6–8)

**Group assigned to the given band**	**Fe^{II}–QUE crystals (cm^{-1})**	**Fe^{II}–QUE NPs (cm^{-1})**
O–H stretching vibration of phenol	3406, 3284	3340 (broad band)
C=O aryl ketonic stretch	1665	1647
C=C aromatic ring stretching band	1606, 1560, 1520, 1450	1599, 1509, 1413
O–H bending of phenol	1380	1355
C–H bond in aromatic hydrocarbon bending (in-plane)	1320, 1010, 997	1315
C–O stretching of aromatic ester (C–O–C)	1261	1269
C–O stretching of aryl ether	1195	1198
C–CO–C stretching and bending in ketone	1165, 1129, 1090	1165
C–H bending of aromatic hydrocarbon (out-of-plane)	1012, 942, 863, 841, 817 785, 637, 595	1012
Fe–O stretching	621 (broad band)	625

Section S4. Supporting References

[1] M. Faria, M. Björnmalm, K. J. Thurecht, S. J. Kent, R. G. Parton, M. Kavallaris, A. P. R. Johnston, J. J. Gooding, S. R. Corrie, B. J. Boyd, P. Thordarson, A. K. Whittaker, M. M. Stevens, C. A. Prestidge, C. J. H. Porter, W. J. Parak, T. P. Davis, E. J. Crampin, F. Caruso, *Nat. Nanotechnol.* **2018**, *13*, 777–785.

Checklist

Minimum Information Reporting in Bio–Nano Experimental Literature

The MIRIBEL guidelines were introduced here: https://doi.org/10.1038/s41565-018-0246-4
The development of these guidelines was led by the ARC Centre of Excellence in Convergent Bio-Nano Science and Technology: https://www.cbns.org.au/. Any updates or revisions to this document will be made available here: http://doi.org/10.17605/OSF.IO/SMVTF. This document is made available under a CC-BY 4.0 license: https://creativecommons.org/licenses/by/4.0/.
The MIRIBEL guidelines were developed to facilitate reporting and dissemination of research in bio–nano science. Their development was inspired by various similar efforts:

- MIAME (microarray experiments): *Nat. Genet.* **29** (2001), 365; http://doi.org/10.1038/ng1201-365
- MIRIAM (biochemical models): *Nat. Biotechnol.* **23** (2005) 1509; http://doi.org/10.1038/nbt1156
- MIBBI (biology/biomedicine): *Nat. Biotechnol.* **26** (2008) 889; http://doi.org/10.1038/nbt.1411
- MIGS (genome sequencing): *Nat. Biotechnol.* **26** (2008) 541; http://doi.org/10.1038/nbt1360
- MIQE (quantitative PCR): *Clin. Chem.* **55** (2009) 611; http://doi.org/10.1373/clinchem.2008.112797
- ARRIVE (animal research): *PLOS Biol.* **8** (2010) e1000412; http://doi.org/10.1371/journal.pbio.1000412
- *Nature*'s reporting standards:
  - Life science: https://www.nature.com/authors/policies/reporting.pdf; e.g., *Nat. Nanotechnol.* **9** (2014) 949; http://doi.org/10.1038/nnano.2014.287
  - Solar cells: https://www.nature.com/authors/policies/solarchecklist.pdf; e.g., *Nat. Photonics* **9** (2015) 703; http://doi.org/10.1038/nphoton.2015.233
  - Lasers: https://www.nature.com/authors/policies/laserchecklist.pdf; e.g., *Nat. Photonics* **11** (2017) 139; http://doi.org/10.1038/nphoton.2017.28
- The "TOP guidelines": e.g., *Science* **352** (2016) 1147; http://doi.org/10.1126/science.aag2359

Similar to many of the efforts listed above, the parameters included in this checklist are **not** intended to be definitive requirements; instead they are intended as 'points to be considered', with authors themselves deciding which parameters are—and which are not—appropriate for their specific study.
This document is intended to be a living document, which we propose is revisited and amended annually by interested members of the community, who are encouraged to contact the authors of this document. Parts of this document were developed at the annual International Nanomedicine Conference in Sydney, Australia: http://www.oznanomed.org/, which will continue to act as a venue for their review and development, and interested members of the community are encouraged to attend.
After filling out the following pages, this checklist document can be attached as a "Supporting Information" document during submission of a manuscript to inform Editors and Reviewers (and eventually readers) that all points of MIRIBEL have been considered.

Supplementary Table 1. Material characterization*

Question	Yes	No
1.1 Are "**best reporting practices**" **available** for the nanomaterial used? For examples, see *Chem. Mater.* **28** (2016) 3535; http://doi.org/10.1021/acs.chemmater.6b01854 and *Chem. Mater.* **29** (2017) 1; http://doi.org/10.1021/acs.chemmater.6b05235	**Not applicable**	
1.2 If they are available, **are they used**? If not available, ignore this question and proceed to the next one.		
1.3 Are extensive and clear instructions reported detailing all steps of **synthesis** and the resulting **composition** of the nanomaterial? For examples, see *Chem. Mater.* **26** (2014) 1765; http://doi.org/10.1021/cm500632c, and *Chem. Mater.* **26** (2014) 2211; http://doi.org/10.1021/cm5010449. Extensive use of photos, images, and videos are strongly encouraged. For example, see *Chem. Mater.* **28** (2016) 8441; http://doi.org/10.1021/acs.chemmater.6b04639	√	
1.4 Is the **size** (or **dimensions**, if non-spherical) and **shape of** the nanomaterial reported?	√	
1.5 Is the **size dispersity** or **aggregation** of the nanomaterial reported?	√	
1.6 Is the **zeta potential** of the nanomaterial reported?	√	
1.7 Is the **density (mass/volume)** of the nanomaterial reported?	**Not applicable**	
1.8 Is the amount of any **drug loaded** reported? 'Drug' here broadly refers to functional cargos (e.g., proteins, small molecules, nucleic acids).	√	
1.9 Is the **targeting performance** of the nanomaterial reported, including **amount** of ligand bound to the nanomaterial if the material has been functionalised through addition of targeting ligands?	√	
1.10 Is the **label signal** per nanomaterial/particle reported? For example, fluorescence signal per particle for fluorescently labelled nanomaterials.	√	
1.11 If a material property not listed here is varied, has it been **quantified**?	√	
1.12 Were characterizations performed in a **fluid mimicking biological conditions**?	**Not applicable**	
1.13 Are details of how these parameters were **measured/estimated** provided?	√	
Explanation for **No** (if needed):		

***Ideally, material characterization should be performed in the same biological environment as that in which the study will be conducted. For example, for cell culture studies with nanoparticles, characterization steps would ideally be performed on nanoparticles dispersed in cell culture media. If this is not possible, then characteristics of the dispersant used (e.g., pH, ionic strength) should mimic as much as possible the biological environment being studied.

Supplementary Table 2. Biological characterization*

**Question**	**Yes**	**No**
2.1 Are **cell seeding details**, including **number of cells plated, confluency at start of experiment,** and **time between seeding and experiment** reported?	√	
2.2 If a standardised cell line is used, are the **designation and source** provided?	√	
2.3 Is the **passage number** (total number of times a cell culture has been subcultured) known and reported?	**Not applicable**	
2.4 Is the last instance of **verification of cell line** reported? If no verification has been performed, is the time passed and passage number since acquisition from trusted source (e.g., ATCC or ECACC) reported? For information, see *Science* **347** (2015) 938; http://doi.org/10.1126/science.347.6225.938	**Not applicable**	
2.5 Are the results from **mycoplasma testing** of cell cultures reported?	√	
2.6 Is the **background signal of cells/tissue** reported? (E.g., the fluorescence signal of cells without particles in the case of a flow cytometry experiment.)	√	
2.7 Are **toxicity studies** provided to demonstrate that the material has the expected toxicity, and that the experimental protocol followed does not?	√	
2.8 Are details of media preparation (**type of media, serum,** any **added antibiotics**) provided?	√	
2.9 Is a **justification of the biological model** used provided? For examples for cancer models, see *Cancer Res.* **75** (2015) 4016; http://doi.org/10.1158/0008-5472.CAN-15-1558, and *Mol. Ther.* **20** (2012) 882; http://doi.org/10.1038/mt.2012.73, and *ACS Nano* **11** (2017) 9594; http://doi.org/10.1021/acsnano.7b04855	√	
2.10 Is characterization of the **biological fluid** (*ex vivo*/*in vitro*) reported? For example, when investigating protein adsorption onto nanoparticles dispersed in blood serum, pertinent aspects of the blood serum should be characterised (e.g., protein concentrations and differences between donors used in study).	**Not applicable**	
2.11 For **animal experiments**, are the ARRIVE guidelines followed? For details, see *PLOS Biol.* **8** (2010) e1000412; http://doi.org/10.1371/journal.pbio.1000412	**Not applicable**	
Explanation for **No** (if needed):		

*For *in vitro* experiments (e.g., cell culture), *ex vivo* experiments (e.g., in blood samples), and *in vivo* experiments (e.g., animal models). The questions above that are appropriate depend on the type of experiment conducted.

Supplementary Table 3. Experimental details*

**Question**	**Yes**	**No**
3.1 For cell culture experiments: are **cell culture dimensions** including **type of well**, **volume of added media**, reported? Are cell types (i.e.; adherent vs suspension) and **orientation** (if non-standard) reported?	√	
3.2 Is the **dose of material administered** reported? This is typically provided in nanomaterial mass, volume, number, or surface area added. Is sufficient information reported so that regardless of which one is provided, the other dosage metrics can be calculated (i.e. using the dimensions and density of the nanomaterial)?	√	
3.3 For each type of imaging performed, are details of how **imaging** was performed provided, including details of **shielding, non-uniform image processing**, and any **contrast agents** added?	√	
3.4 Are details of how the dose was administered provided, including **method of administration, injection location, rate of administration,** and details of **multiple injections**?	**Not applicable**	
3.5 Is the methodology used to **equalise dosage** provided?	√	
3.6 Is the **delivered dose** to tissues and/or organs (in vivo) reported, as % injected dose per gram of tissue (%ID g^{-1})?	**Not applicable**	
3.7 Is **mass of each organ/tissue measured** and **mass of material** reported?	**Not applicable**	
3.8 Are the **signals of cells/tissues with nanomaterials** reported? For instance, for fluorescently labelled nanoparticles, the total number of particles per cell or the fluorescence intensity of particles + cells, at each assessed timepoint.	√	
3.9 Are **data analysis details**, including **code used** for analysis provided?	√	
3.10 Is the **raw data** or **distribution of values** underlying the reported results provided? For examples, see *R. Soc. Open Sci.* **3** (2016) 150547; http://doi.org/10.1098/rsos.150547, https://opennessinitiative.org/making-your-data-public/, http://journals.plos.org/plosone/s/data-availability, and https://www.nature.com/sdata/policies/repositories	√	
Explanation for **No** (if needed):		

* The use of protocol repositories (e.g., *Protocol Exchange* http://www.nature.com/protocolexchange/) and published standard methods and protocols (e.g., *Chem. Mater.* **29** (2017) 1; http://doi.org/10.1021/acs.chemmater.6b05235, and *Chem. Mater.* **29** (2017) 475; http://doi.org/10.1021/acs.chemmater.6b05481) are encouraged.
